# Investigation of suddenly expanded flows at subsonic Mach numbers using an artificial neural networks approach

**DOI:** 10.1371/journal.pone.0276074

**Published:** 2022-10-26

**Authors:** Jaimon Dennis Quadros, Chetna Nagpal, Sher Afghan Khan, Abdul Aabid, Muneer Baig

**Affiliations:** 1 Fluids Group, School of Mechanical Engineering, Istanbul Technical University, Istanbul, Turkey; 2 Faculty of Engineering, Al Reef Institute of Logistics and Applied Technology, Abu Dhabi Vocational Education and Training Institute (ADVETI), Abu Dhabi, United Arab Emirates; 3 Department of Mechanical Engineering, Kulliyyah of Engineering, International Islamic University Malaysia, Selangor, Malaysia; 4 Department of Engineering Management, College of Engineering, Prince Sultan University, Riyadh, Saudi Arabia; Tianjin University, CHINA

## Abstract

The purpose of this study is to explore two concepts: first, the use of artificial neural networks (ANN) to forecast the base pressure (*β*) and wall pressure (*ω*) originating from a suddenly expanded flow field at subsonic Mach numbers. Second, the implementation of Garson approach to determine the critical operating parameters affecting the suddenly expanded subsonic flow process in the subsonic range. In a MATLAB environment, a network model was constructed based on a multilayer perceptron with an input, hidden, and output layer. The network input parameters were the Mach number (*M*), nozzle pressure ratio (*η*), area ratio (*α*), length to diameter ratio (*γ*), micro jet control (*ϵ*), and duct location to length ratio (*δ*). The network output included two variables; base pressure (*β*) and wall pressure (*ω*). The ANN was trained and tested using the experimental data. The experimental results found that micro-jet controls were successful in increasing the base pressure for low Mach numbers and high nozzle pressure ratios. It was also found that the wall pressure was same for with and without micro jet control. The ANN predicted values agreed well with the experimental values, with average relative errors of less than 5.02% for base pressure and 6.71% for wall pressure. Additionally, with a relative significance of 32% and 43%, the nozzle pressure ratio and duct location to length ratio had the highest influence on the base pressure and wall pressure, respectively. The results demonstrate that the ANN model is capable of accurately predicting the pressure results, enabling theoretical foundation for research into pressure distribution in aerodynamic systems.

## 1. Introduction

A rapid increase in the area gives rise to flow separation and reattachment. Despite great efforts, high-speed separated flows are not totally realized. Due to the complexity of fluid dynamic events, these flows are difficult to anticipate accurately. This includes the existence of shock waves, expansion waves, and pressure gradients in the flow properties of the fluid [1–4]. The departing shear layer creates recirculation flow zones with sub-atmospheric pressure at the base at high Reynolds numbers. These recirculation zones can be extremely turbulent and highly compressible [2]. Understanding the fluid dynamic behavior in the near-field region behind axi-symmetric structures is vital for predicting the high-speed rocket and aerodynamic vehicle performance. At transonic and supersonic Mach numbers, the emergence of a low-pressure recirculation zone operating near the rear-looking base of the blunt bodies which are of cylindrical shape, can significantly impact the base drag values. It is discovered that the base drag coefficients at transonic Mach numbers account for approximately 60% of the overall drag of aerodynamic vehicles [5, 6].

Korst [7] was the first to examine the base pressure (*β*) problem in transonic and supersonic flow regimes, when the external flow at the base and wake was sonic and supersonic, respectively. By assessing the relation between the neighboring jet and the dissipative shear flow in the wake, a physical model was established. Further research found that the use of passive techniques in controlling *β* in separated flows were effective and has been studied extensively [8–10]. In the case of passive control, the flow is regulated by altering the geometry of the model. As a result, passive control mechanisms such as grooved cavities, ribs, step bodies, a boat tail, and a locked vortex were utilized to regulate the fluid flow in the wake zone. It was established that the *β* level is a function of the inertia level at the nozzle exit, the level of expansion, possible relaxation available at the nozzle exit, and length to diameter ratio (*γ*). Additionally, the reattachment length position is dictated by the area ratio (*α*), expansion level (*η*), and Mach number (*M*). The literature concluded that it is case-sensitive, and one must determine on a case-by-case basis.

Another approach employed by researchers to regulate *β* was the use of active control. These studies deal with an active control mechanism wherein, microjets are employed to manage *β* in high-speed flows at varying Mach numbers. One of the key difficulties with active control is the need for additional energy to engage the control mechanism. Similarly, the abruptly expanded flow in a cylindrical duct was explored for the scenario of noise generation and *β* [11], which was minimum due to the attached flow and proportional to the ratio of duct to nozzle exit area (*α*). Experiments were carried out by Khan and Rathakrishnan [1–4, 12] to determine the behavior of micro jets influencing over, under, and proper expansion in order to regulate *β* in abruptly expanded axisymmetric ducts. Mach numbers (*M*) of 1.25, 1.3, 1.48, 1.6, 1.87, 2.2, and 2.58 were considered in the study. For *M* = 2.58, the largest rise in *β* was experienced i.e., 152%. The micro jets did not have any adverse influence on pressure distribution along the wall. It was also observed that *η* played a critical role in governing the *β* fluctuation for both instances, i.e., with and without microjet control. Furthermore, experiments were performed on underexpanded and properly expanded cases in the duct at *M* = 1.25 and *α* = 1.6. It was seen that the flow turned oscillatory when *η* and *α* were regulated separately for both with and without control. Several other experimental research works were carried out by using an active/passive control system to control flow separation [13–18]. Furthermore, these experimental works were validated using the computational fluid dynamics (CFD) technique by many researchers. For instance, numerical simulation of flows via a convergent divergent (C-D) nozzle at *M* = 2.6 was performed [19]. To examine the effect of microjets, numerical simulations were conducted using the *k-ε* turbulence model [20] and the ANSYS FLUENT code. The flow field in a C-D nozzle was investigated using a density-based solver [21]. Besides this, splitter plate effects were employed to minimize drag and regulate the flow fields in rectangular bluff bodies [22] and D-shaped bluff bodies (non-circular cylinders) [23]. All the numerically simulated results agreed well with experiments.

Apart from the above works, various studies pertaining to high speed flow have been conducted by employing fuzzy logic, neural networks, or optimization, a type of soft computing study, which has given an all new direction to these kinds of problems. Soft computing is an emerging approach that matches the human mind’s remarkable capacity to target and study in the face of ambiguity and imprecision. It incorporates a variety of computer models, such as fuzzy set theory, neural networks, optimization approach, approximation reasoning, clustering, classification, regression, etc. For the first time, Wang et al. [24] applied a physics-informed machine learning technique to predict high speed Mach flows. This technique efficiently modelled the Reynolds averaged Navier Stokes (RANS) turbulent model over a flat plate boundary layer through direct numerical simulation. Nott et al. [25] used genetic algorithms, neural networks, and Proportional Integral Differential (PID) to regulate the flow control parameters in the supersonic regime. A genetic algorithm was used to optimize the controller based on a neural network (NN) model. The proposed method was modified and proved to be in excellent agreement with the PID data. Fan et al. [26] modelled the aerodynamic data using a support vector machine classification technique. They compared the ANN and support vector machine techniques using data from a novel prototype mixer for engine combustors and a multiple hole pressure probe calibration. Dupuis and Jouhaud [27] used a machine-learning-based surrogate model to forecast the aerodynamic data for transonic flows. In their study, the surrogate model was used to split the training samples’ solutions into homogeneous clusters and to partition the parameter space according to the form of the solution. The suggested framework readily predicted flow regime detection and global accuracy. Similar efforts on the application of computational methods to the detection and modeling of aerodynamic flows are documented in [28–30]. Afzal et al. [31] generated a back propagation neural network model (BPNN) for flows that were abruptly expanded. Various flow control parameters such as, *M*, *γ*, *α*, and *η*, were identified, and their influence on *β* and *ω* was examined. The massive experimental data was visualized using heat maps. Six BPNN models were developed on the basis of input and output possibilities to predict *β* and *ω*. The data visualization revealed that *η* greatly influenced *β*. BPM 5 and 6 successfully forecasted the highly non-linear *β* and *ω*. Jaimon and Khan [32] developed a predictive model for *β* in the abruptly expanded flow stream using the artificial neural networks (ANN) approach and computational fluid dynamics (CFD). An experimental test rig consisting of a nozzle and abruptly expanded duct developed results for *β* and validated the CFD results. The hidden layer in the ANN architecture comprised of eight neurons. The ANN accurately predicted *β* with a regression coefficient *R*^*2*^ less than 0.99 and a root mean square error (*RMSE*) of 0.0032. The *β* and *ω* for flows from nozzle into the enlarged duct was investigated in detail by Afzal et al. [33]. To augment the *β*, microjets with active flow control were used. Experiments were conducted for *M* ranging from 1 to 3, and *η* ranging from 3 to 11. The K-means algorithm that clusters massive data, revealing important facts and patterns about pressure behavior was used for the analysis. Also, both pressures were modeled using a random forest classification technique. K-means clustering indicated that a significant proportion of *β* falls in the lower range. The random forest technique was observed to be an extremely useful tool for estimating *β*, *ω*, and other highly non-linear data.

Studies were conducted to determine the optimal base pressure in a nozzle flow via an abrupt expansion duct with and without microjet control. Among these investigations, the design of experiments (DoE) and response surface methodology (RSM) technique enabled determining the influence of factors on fluid flow process control. One such effort by Jaimon et al. [34] used the RSM based central composite design (CCD) and Box-Behnken design (BBD) to develop a non-linear response equation that would predict *β* for different inputs at varying levels. The input parameters were *M*, *γ*, *α*, and *η*, and were set to low, medium, and high levels, based on trial experiments. The response equation accurately predicted *β* with an average percentage error of ≤ 5.50%. The proposed model was tested for significance using analysis of variance. Aabid and Khan [35] optimized *β* using the DoE approach. The experimental parameters of the study were *α* = 3.24, *γ* ranging from 10 to 1, and *M* = 1.87, 2.2, and 2.58, respectively. Additionally, the CFD technique that implemented the *k-ε* turbulent model, was used to validate the experimental data. The results showed that *γ* impacted *β* significantly when compared to the other parameters. Aabid et al. [36] used experimental and optimization techniques to determine *β* and *ω* for micro jets located at (i) 90° intervals of the base; (ii) located at 0.5D of the wall; (iii) located both at the base and wall. The parameters of the study were *M* = 1.87, 2.2, and 2.58, *γ* = 10 to 1, *and η* = 3, 5, 7, 9, and 11. A DoE based L_9_ orthogonal array was used for planning the experiments. The results were optimized through analysis of variance, response surface, and regression equations. The results indicated that for a given limitation, one may determine the duct length of the pipe that would result in the maximum rise or fall of pressure in the base region.

A thorough evaluation of literature on high speed and compressible flow indicates that including experiments, many studies examined *β* in high speed flows using different techniques such as, RSM [34, 35], ANN [31–33], and CFD [20–22]. Most of these studies were applied to Mach flows in the sonic and supersonic range i.e., (*M* ≥1). Analysis of pressure fluctuation in subsonic Mach flow range i.e., (*M*<1) has not been explored yet. Also, neural network modelling and critical parameter identification using the Garson approach for these types of complex flows is also new. These approaches are nowhere described previously for subsonic Mach flows. Considering the existing gap, the current study analyzed *β* and *ω* for suddenly expanded subsonic flows using experiments and ANN. The flow Mach numbers selected in this study were in the subsonic range i.e., (0.30 ≤ *M* ≤ 0.90).

## 2. Experimental set up and operating parameters

**Fig 1:**
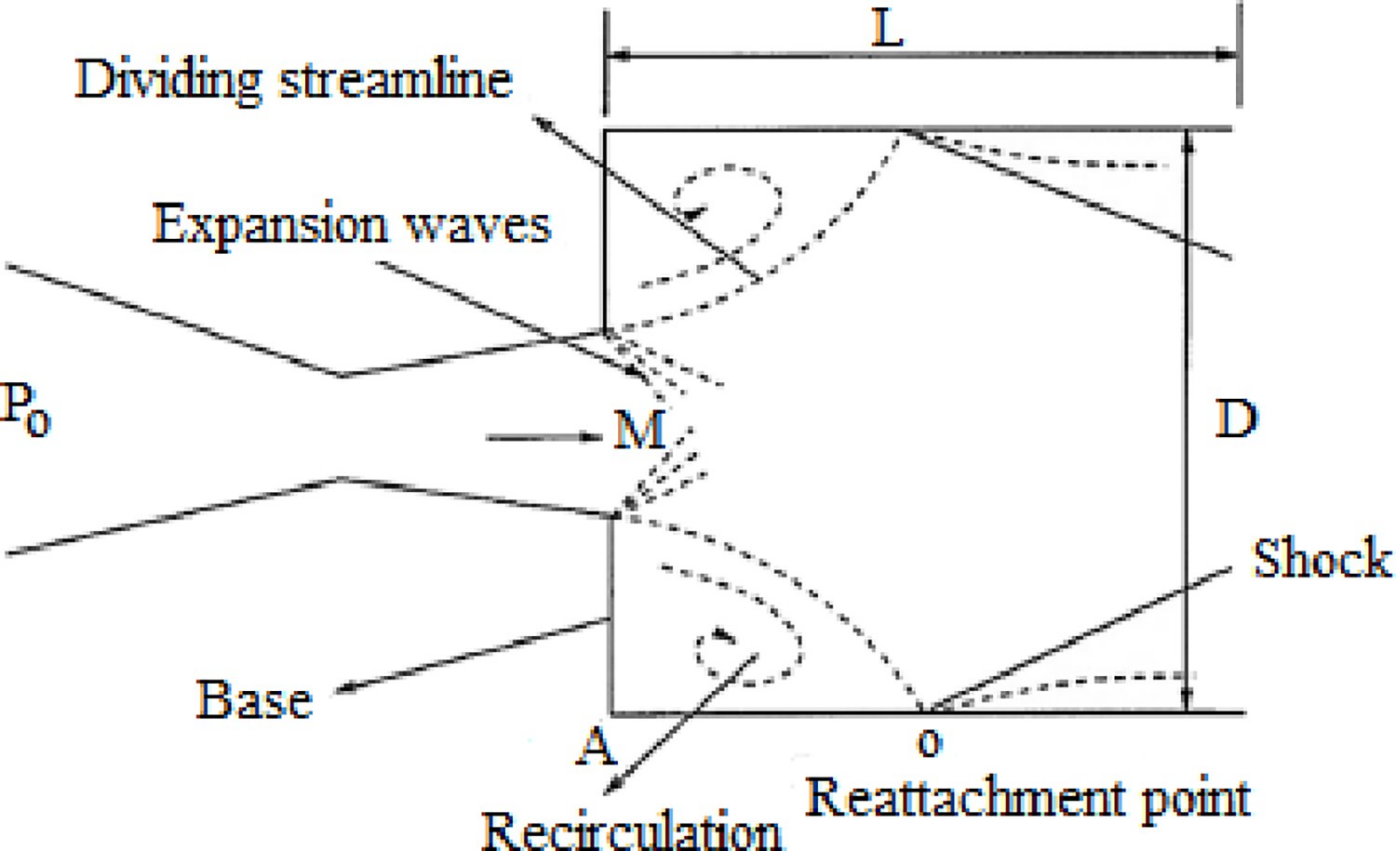
Suddenly expanded flow field [1, 2].

**Fig 2:**
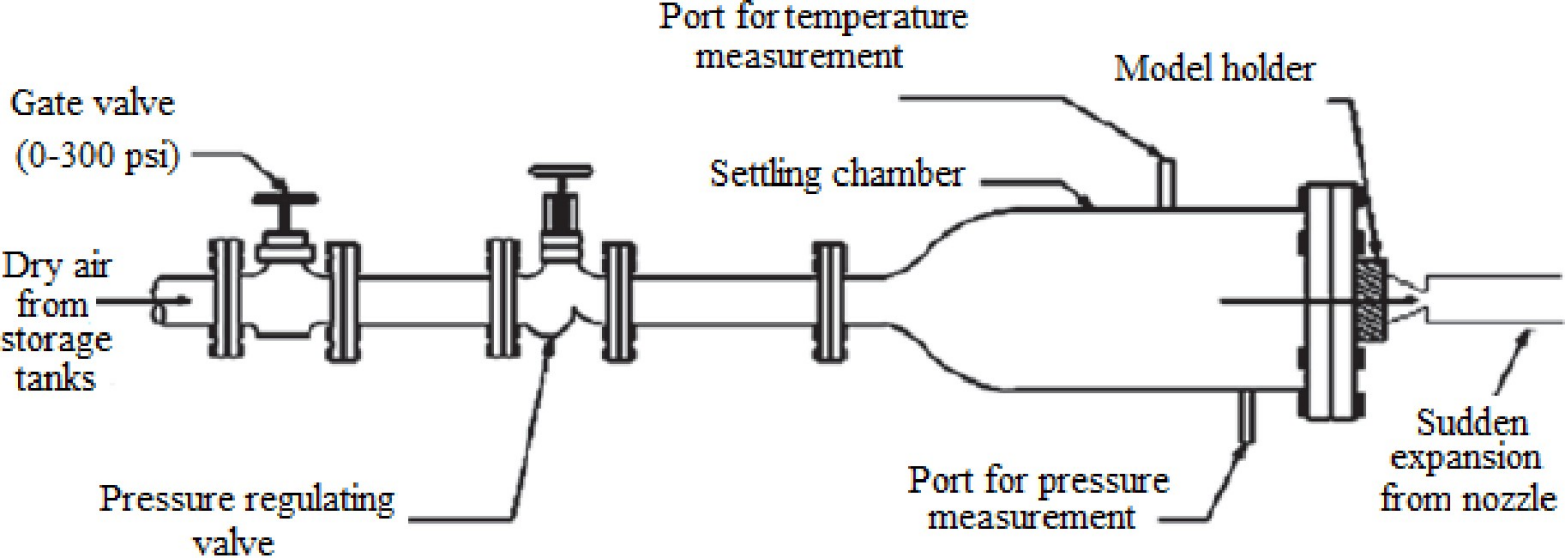
Test jet apparatus [8].

**Fig 3:**
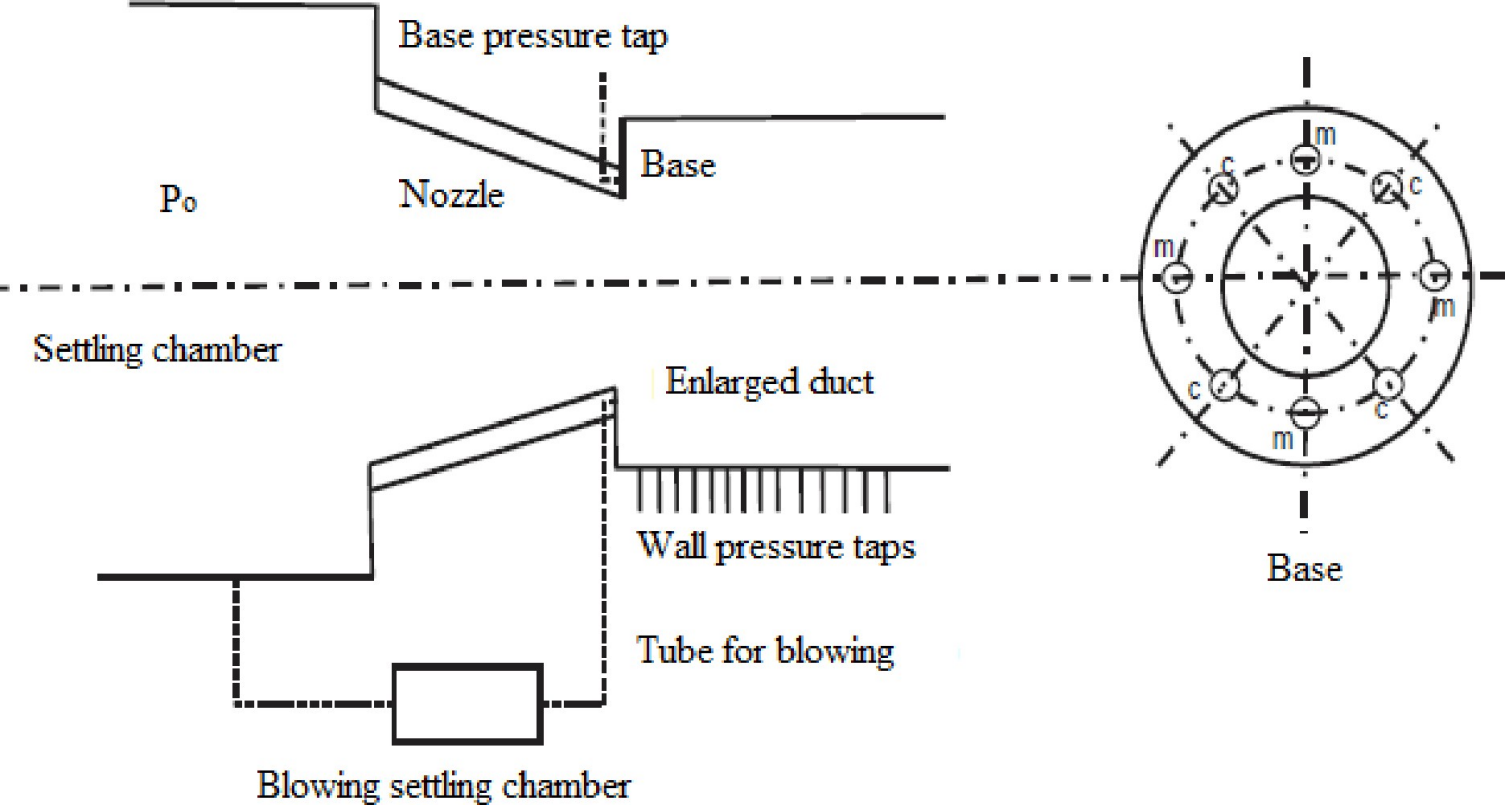
Schematic of the experimental set up [16].

The sudden expansion flow characteristics such as the recirculation zone, reattachment point, and expansion waves are investigated experimentally, and presented in Fig 1. The schematic depiction of the test jet facility and the experimental set up is shown in Figs 2 and 3, respectively. Two key components form the experimental setup: a flow apparatus consisting of compressors, and storage reservoirs, and an open jet apparatus. Air at high pressure is supplied to the settling chamber via a control segment that generally consists of pressure regulating valve and throttle valve. The throttle is attached to a mixing tube that delivers the settling chamber with the air it requires. The terminus of the settling chamber is attached to the nozzle through a slot holder. This arrangement consists of a tiny pipe-like extension that incorporates an O-ring to prevent leakage. Due to the rapid expansion of the discharge from the nozzle, three pipes with diameters (D) of 16, 18, 21.80, and 25 mm have been attached directly to the nozzle exit. These four diameters correlate to *α* = 2.56, 3.25, 4.75, and 6.25, respectively, which are used in the current investigation. All nozzles have a fixed exit diameter of d = 10 mm. The settling chamber provides the requisite *η* of 1.2, 1.5, and 1.8 for the current investigation, via stagnation pressure (P_o_). Additionally, laboratory air pressure was measured during the experiment using the barometer. Table 1 summarizes the ranges for the operating conditions at the inlet of the abruptly expanded subsonic flow process.

**Table 1:** Details of operating conditions for the ANN model.

Operating conditions	Notations	Values
Subsonic Mach number	M	0.3, 0.4, 0.5, 0.6, 0.7, 0.8, 0.9
Nozzle pressure ratio	*Η*	1.2, 1.5, 1.8
Area ratio	*Α*	2.56, 3.25, 4.75, 6.25
Length to diameter ratio (L/D)	*Γ*	2, 4, 6, 8, 10
Micro-jet control	*ϵ*	1 (without control), 2 (with control)
Duct location to length ratio	*Δ*	0.3, 0.5. 0.7, 0.9

For the current investigation, nozzles with exit Mach values of 0.3, 0.4, 0.5, 0.6, 0.7, 0.8, 0.9 were manufactured. The nozzles were designed using isentropic relations to optimize the nozzle exit Mach number [Genick, [37]]. The duct was constructed using a 1.5 mm thick brass tube. For the case of *α* = 3.25, during the initial stage of the experiment, the duct diameter was 18 mm and the length was 180 mm, i.e., (L = 10D). Following that, the duct was dissected sequentially at L = 9D, 8D, 7D, 6D, 5D, 4D, and 3D after collecting the needed *β* measurements for the previous L/D. In this experiment, the *β* was established by placing pressure taps at appropriate locations (see Fig 3). The holes were appropriately placed to facilitate pressure taps. Around eight holes were made round the flange that connected the nozzle and duct to measure the *β*. These taps were made of stainless steel and had a diameter of 0.5 mm. These taps were placed perpendicularly into the holes bored in the flange. Pressure ports were used to connect the pressure taps to the pressure transducer. Pressure measurements were taken using a (PSI) 9016 transducer with a pressure range of 0 to 300 psi. It contained sixteen channels that displayed mean values of *β* at the rate of 250 samples/sec. The transducer was connected to a PC running with the LabVIEW software. The transducer had an accuracy range of 0.15%. The experimental readings for this investigation were obtained via repeated tests. The uncertainty analysis was performed as per Khan and Rathakrishnan [3, 4]. A steady room temperature with deviation of around ±0.5°C was maintained. Despite notable fluctuations, the pressure data recordings were accurate to within ±2% and repeatable within ±3%.

## 3. ANN modelling approach

An artificial neural network (ANN), alternatively referred to as a "neural network," is a mathematical or computational model influenced by the way the human nervous system works. The ANN is more effective than parametric techniques and is capable of identifying, forecasting, and solving complex problems. Numerous reasons exist stating ANN to be an effective tool for modeling basic fluid flow problems [38].

Firstly, it possesses an extraordinary capacity to accurately identify the underlying relationship among any set of inputs and outputs without requiring a physical model or even knowledge about the intrinsic behavior of the system. This ability is largely irrespective of the complexity involved, such as non-linearity, multiple variables, and levels. This critical capacity is referred to as pattern recognition, and it is acquired through the training process. Second, the approach is intrinsically fault-tolerant, owing to the vast volume of networking units doing enormous and simultaneous data processing. Thirdly, the capacity of ANNs to train, enables the approach to adapt to the variations in the parameters. This capability permits the ANN to handle time-dependent dynamic modeling as well.

A typical neural network (NN) is composed of three layers: an input layer, a hidden layer, and an output layer. The layout of a typical neuron in a neural network referred to as a multilayer perceptron is composed of a single or several hidden layers that are accountable for the network outputs’ performance. The training procedure for neural networks is based on the adjustment of the connected weights and biases via the learning technique. To generate the neuron output, the weighted aggregate of the inputs is passed through a non-linear function. The NN model is described using the Eq (1) [38]

$${Net}_{k}=\sum_{j=1}^{j=n}W_{i,jk}\times x_{j}+b1_{k}$$ (1)

Similarly, the NN output is given by the Eq (2)

$${Out}_{Net}=PURELIN[W_{o,sk}\times TANSIG(W_{i,jk}x_{j}+b1_{k})]+b2_{s}$$ (2)

where, *x*_1_, *x*_2_………,*x*_*j*_ are referred as input signals; *W*_*i*,11_, *W*_*i*,21_,……,*W*_*i*,*jk*_ are the weights of the input neuron *k*, respectively; *W*_*o*,11_, *W*_*o*,21_,…….,*W*_*o*,*sk*_ are the weights of the output neuron *s*, respectively; *b*1_*k*_ and *b*2_*s*_ are the biases. The present NN model implemented a hyperbolic tangent sigmoid transfer function (TANSIG) between the input and hidden layer, and a linear transfer function (PURELIN) for the output layer. The hyperbolic tangent function is implemented because it has a greater slope means that show a greater response for a small deviation in the input variables. The network output for *β* is expressed as per [39]

$${Out}_{Net1}=\beta =PURELIN[W_{o,sk}\times TANSIG(W_{i,jk}x_{j}+b1_{k})]+b2_{s}$$ (3)

$${Out}_{Net1}=\beta =\sum_{k=1}^{k=m}W_{o,sk}\left[\frac{2}{1+exp(-2\times (\sum_{j=1}^{j=n}W_{i,jk}\times x_{j}+b1_{k}))}-1\right]+b2_{s}$$ (4)

**Fig 4:**
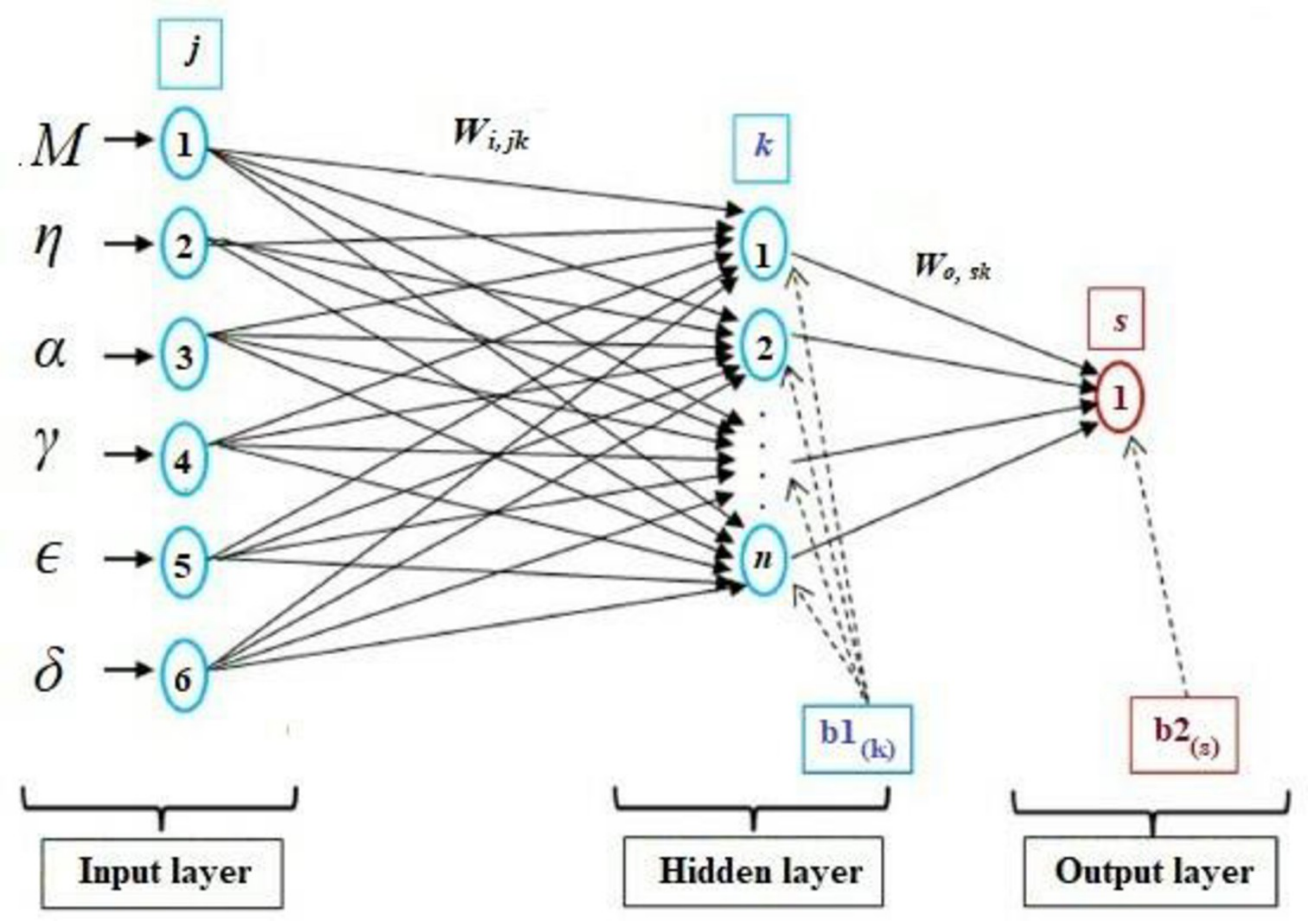
ANN model with input, hidden, and output layers.

In a similar way, the network output for *ω* is expressed as

$${Out}_{Net2}=\omega =PURELIN[{W^{*}}_{o,sk}\times TANSIG({W^{*}}_{i,jk}x_{j}+b{1^{*}}_{k})]+b{2^{*}}_{s}$$ (5)

$${Out}_{Net2}=\omega =\sum_{k=1}^{k=m}{W^{*}}_{o,sk}\left[\frac{2}{1+exp(-2\times (\sum_{j=1}^{j=n}{W^{*}}_{i,jk}\times x_{j}+b{1^{*}}_{k}))}-1\right]+b{2^{*}}_{s}$$ (6)

where, ‘*j*’, ‘*k*’, and ‘*s*’ correspond to input, hidden, and output layers, respectively, and ‘*n*’, ‘*m*’, and ‘*l*’ refer to neurons in the input, hidden, and output layers. *W*_*i*_, *W*_*o*_, W*_i_, W*_o_, and *b*1_*k*_, *b*2_*s*_, *b*1*_k_, *b*2*_s_ refer to weights and biases. Fig 4 illustrates the construction of the ANN used to represent the experimental setup for abruptly expanded subsonic flow process with input and output parameters. For modeling purposes, six process variables were considered: *M*, *η*, *α*, *γ*, micro jet control (*ϵ*), and duct location to length ratio (*δ*) and, as well as two response variables i.e., *β* and *ω*. Following the establishment of the NN architecture, the experiment inputs and outputs were normalized between -1 and +1 using Eq 7 as per [39].

$${val}^{nor}=\frac{{val}_{i}-{val}_{min}}{{val}_{max}-{val}_{min}}(y_{max}-y_{min})+y_{min}$$ (7)

Here, *val*^*nor*^ represents value which is normalized, *val*_*i*_ is the real input or output, *y*_*max*_ and *y*_*min*_ are +1 and -1, respectively, *val*_*max*_ and *val*_*min*_ are the maximum and minimum values of the experimental inputs and outputs, respectively.

**Fig 5:**
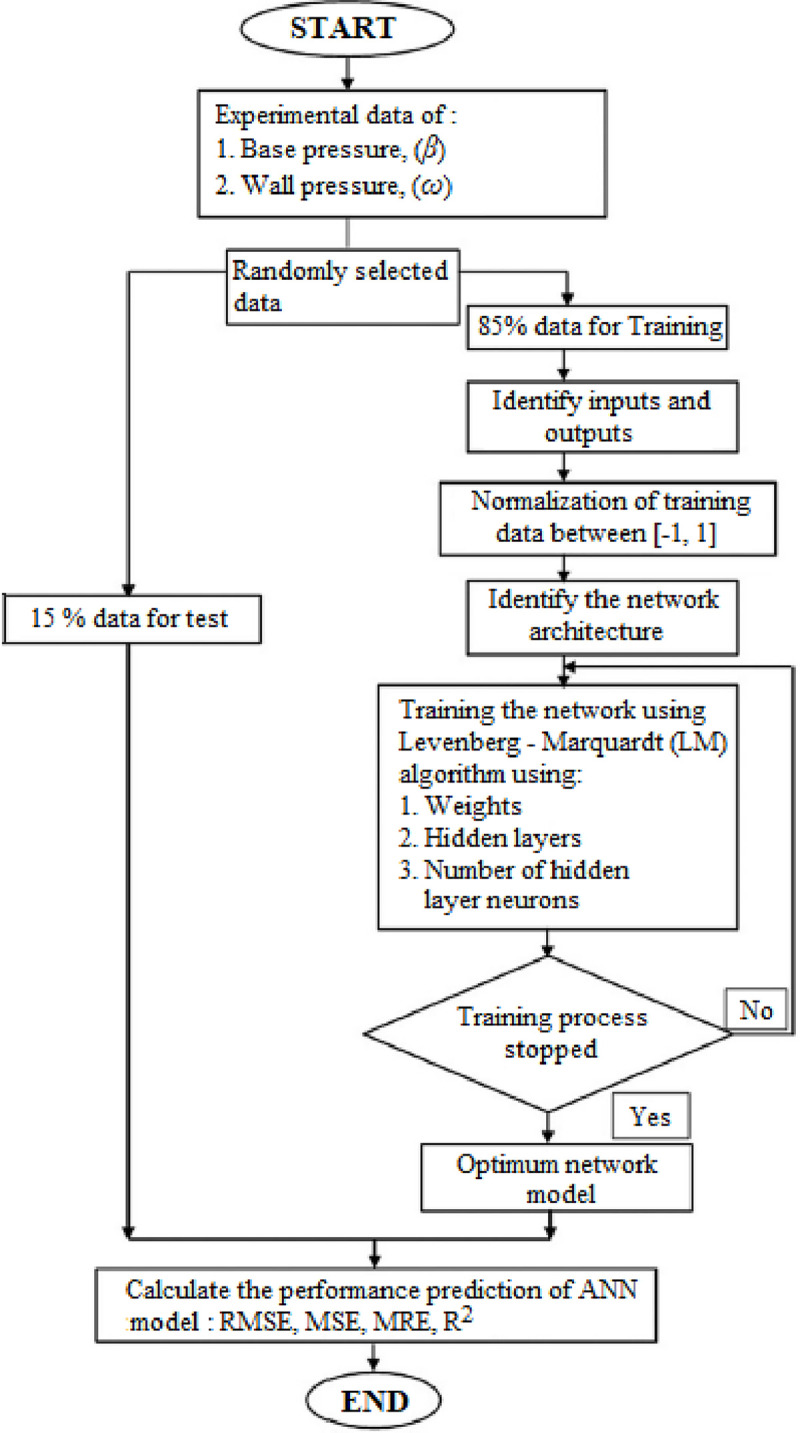
Flowchart for ANN training.

A feed-forward ANN was chosen for this investigation, using a (TANSIG) transfer function in the hidden layer and a linear transfer function in the output layer. The Levenberg-Marquardt technique was used to change the weight coefficients during the training phase. Trial and error method were employed to determine the neurons in the hidden layer. Fig 5 shows the stages required for training the ANN to forecast the *β* and *ω* in the expanded flow process. Whenever any of the following situations occur, the training process is instantly stalled:

- Epochs reached to a maximum.
- Minimum performance i.e., goal = 0 is achieved.
- The gradient of performance is less than (min_grad = 1×10^−7^).

Mean Squared error (*MSE*), root mean square error (*RMSE*), regression coefficient (*R*^*2*^), and mean relative error (*MRE*) have been used to determine the performance prediction of the NN model, given by the Eqs (8, 9, 10, and 11) [39].


$$MSE=\frac{1}{N}\times \sum_{j}{\left(Q_{j,exp}-Q_{j,ANN}\right)}^{2}$$ (8)

$$RMSE=\left(\sqrt{\frac{1}{N}\times \sum_{j}{\left(\left|Q_{j,ANN}-Q_{j,exp}\right|\right)}^{2}}\right)$$ (9)

$$R^{2}=1-\left(\frac{\sum_{j}{\left(Q_{j,ANN}-Q_{j,exp}\right)}^{2}}{\sum_{j}{\left(Q_{j,exp}\right)}^{2}}\right)$$ (10)

$$MRE\;(\%)=\frac{1}{N}\sum_{j}|\frac{\left(Q_{j,exp}-Q_{j,ANN}\right)}{\left(Q_{j,exp}\right)}|\times 100$$ (11)

Here, *Q*_*j*,*ANN*_ represents the NN model predicted value, *Q*_*j*,*exp*_ represents the actual experimental value, and *N* refers to the data number. The current study employed an experimental dataset of 75 input-output pairs to create an ANN model capable of predicting the flow process in terms of *β* and *ω*. Experimental data collected under real operational settings were used to verify the NN model.

## 4. Results and discussions

### 4.1. Base pressure (β) analysis

The purpose of this study is to determine the effect of dynamic control on base pressure (*β*). Additionally, the study will look at the effect of the use of control techniques on the flow growth in the expanded duct. The back-pressure normalized the experimental data acquired in the duct, such as the base pressure (*β*) and static wall pressure (*ω*). When the area of the duct abruptly changes, the *β* is very less (i.e., lower than the atmospheric pressure). The abrupt rise in area leads to flow divergence and reconnection with the duct wall, followed by the formation of a boundary layer (see Fig 1). The Mach numbers (*M*) used in the testing ranged from 0.3 to 0.9. Experiments were undertaken for *η* = 1.2, 1.5, and 1.8. The range of inertia values indicates that the flow exiting from the nozzles was under-expanded.

**Fig 6:**
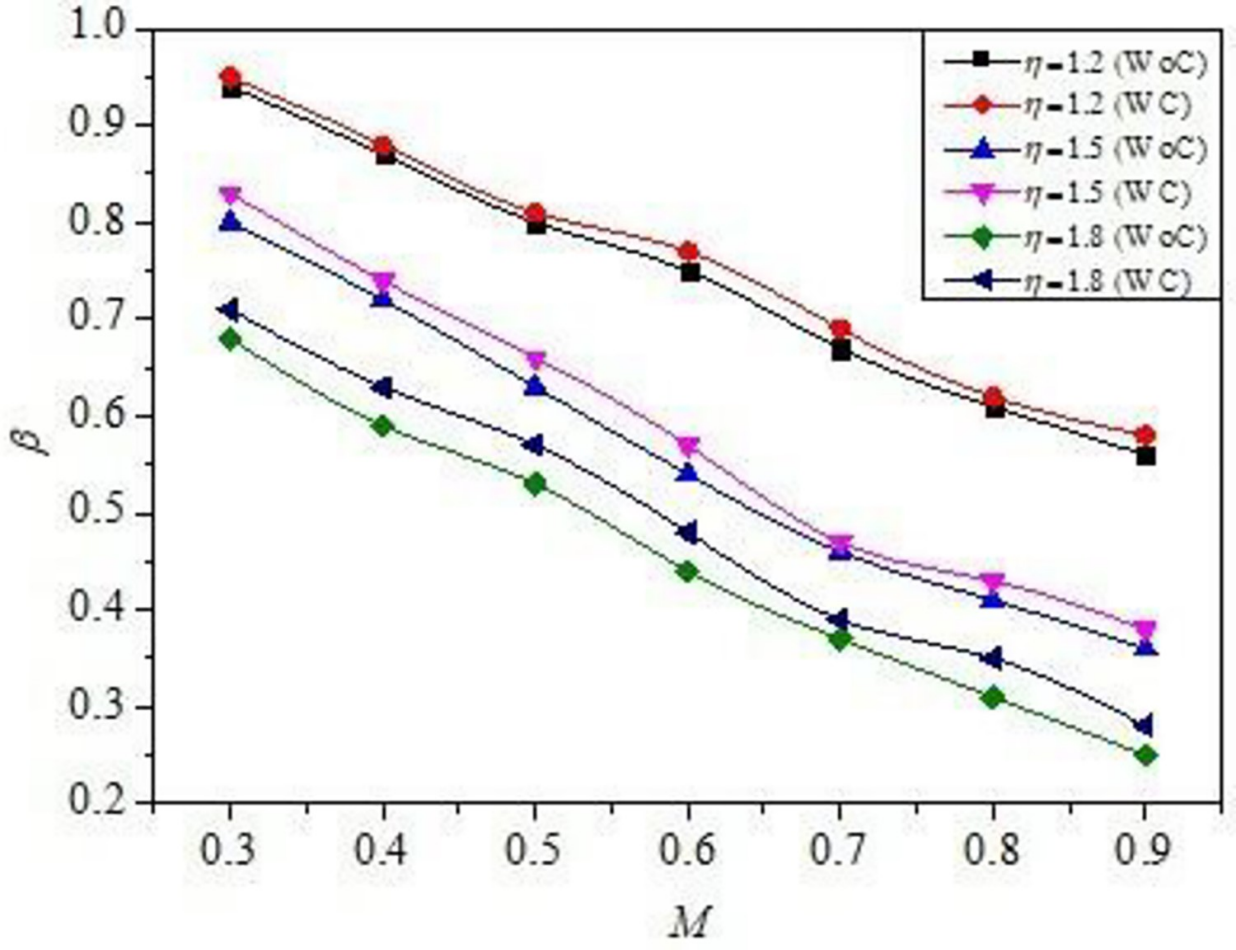
Variation of *β* w.r.t *M* without control (WC) and with control (WC) for different *η =* 1.2, 1.5, and 1.8 for fixed *α* = 4.75 and *γ* = 8.

**Fig 7:**
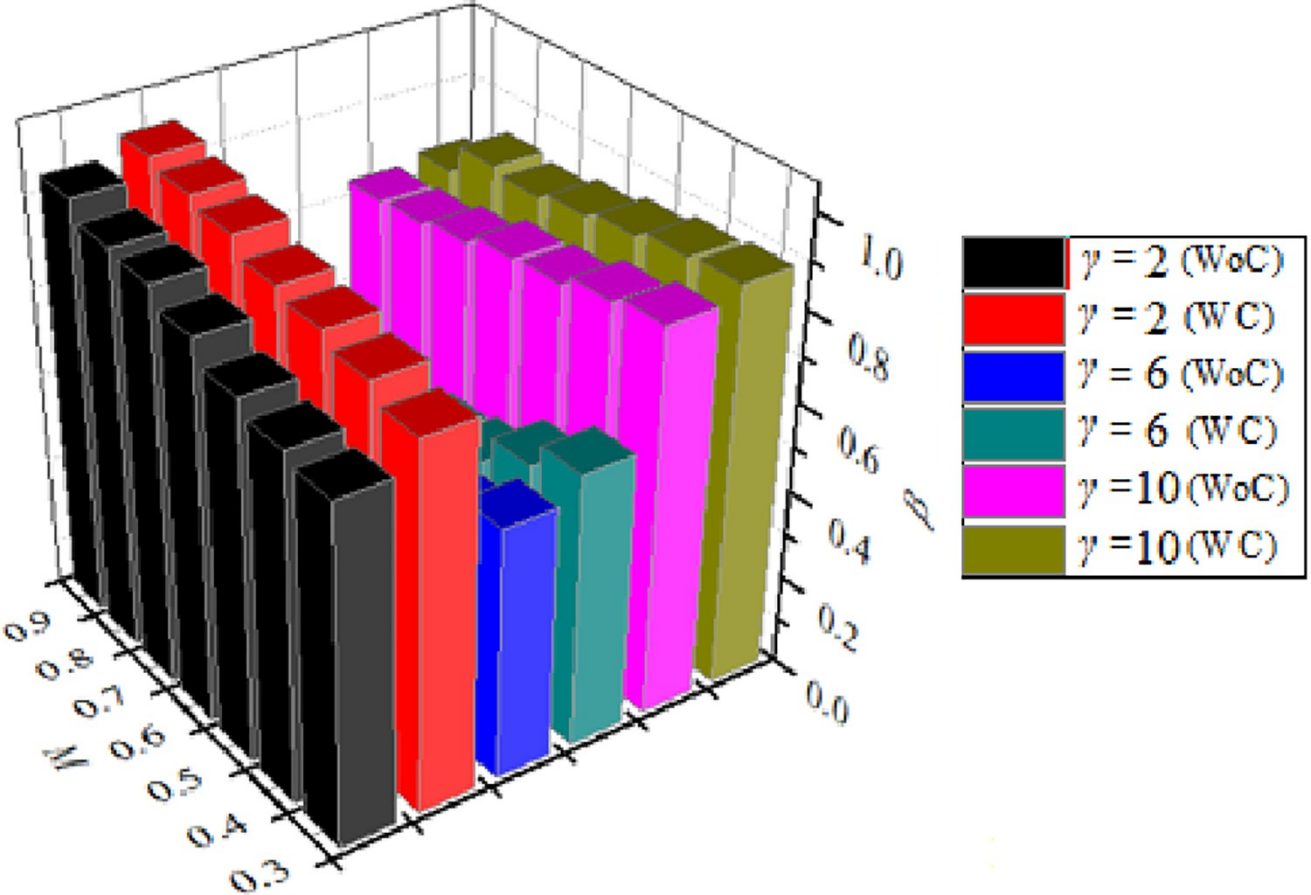
Variation of *β* w.r.t *M* without control (WC) and with control (WC) for different *γ =* 2, 6, and 10 for fixed *α* = 4.75 and *η* = 1.5.

The *β* results for all the *M* values are shown in Fig 6, for the *η* = 1.2, 1.5,1.8, *α* and *γ* were constant at 3.25 and 8, respectively. For the current set of nozzles, the experiments were undertaken for the under-expanded case. Thus, we propose to investigate the efficiency of microjet control (WC) when an unfavorable pressure gradient is present. The purpose of this study was to evaluate the microjet control performance, and to model the mixing process in the combustion chamber. Under-expanded jets are desired at the initial stages of mixing. Following this, the under-expanded nozzle become correctly expanded as the mixing intensifies. From Fig 7, without control (WoC), the flow is under-expanded, and so expands, resulting in a steady drop in the *β*. When the control mechanism (WC) is activated in these conditions, the *β* increases under the existence of a favorable pressure gradient. According to previous studies [31, 33], this trend of decreasing *β* persists until the value of *M* reaches 1.25; however, as the inertia level i.e. *η* increases progressively, the pattern reverses at M = 1.3. Later on, in the *M* range of 1.3–1.48, a further reduction in *β* is observed. Additionally, when the nozzles are not expanded sufficiently, the sudden expansion flow creates a recirculation zone at the nozzle-duct interface. Increased values of *M* strengthen the recirculation zone. Suction created by higher *M* values tends to establish a strong recirculation zone, resulting in the production of low *β* [16, 17]. Also, increased values of *η* result in the formation of a small recirculation zone. This is mostly due to the fact that the suction at the base is very strong in contrast to the suction at lower values of *η*, and the breaking of large-scale vortices into small-scale eddies does not occur completely, resulting in low *β* for higher *η* values (Fig 6).

**Fig 8:**
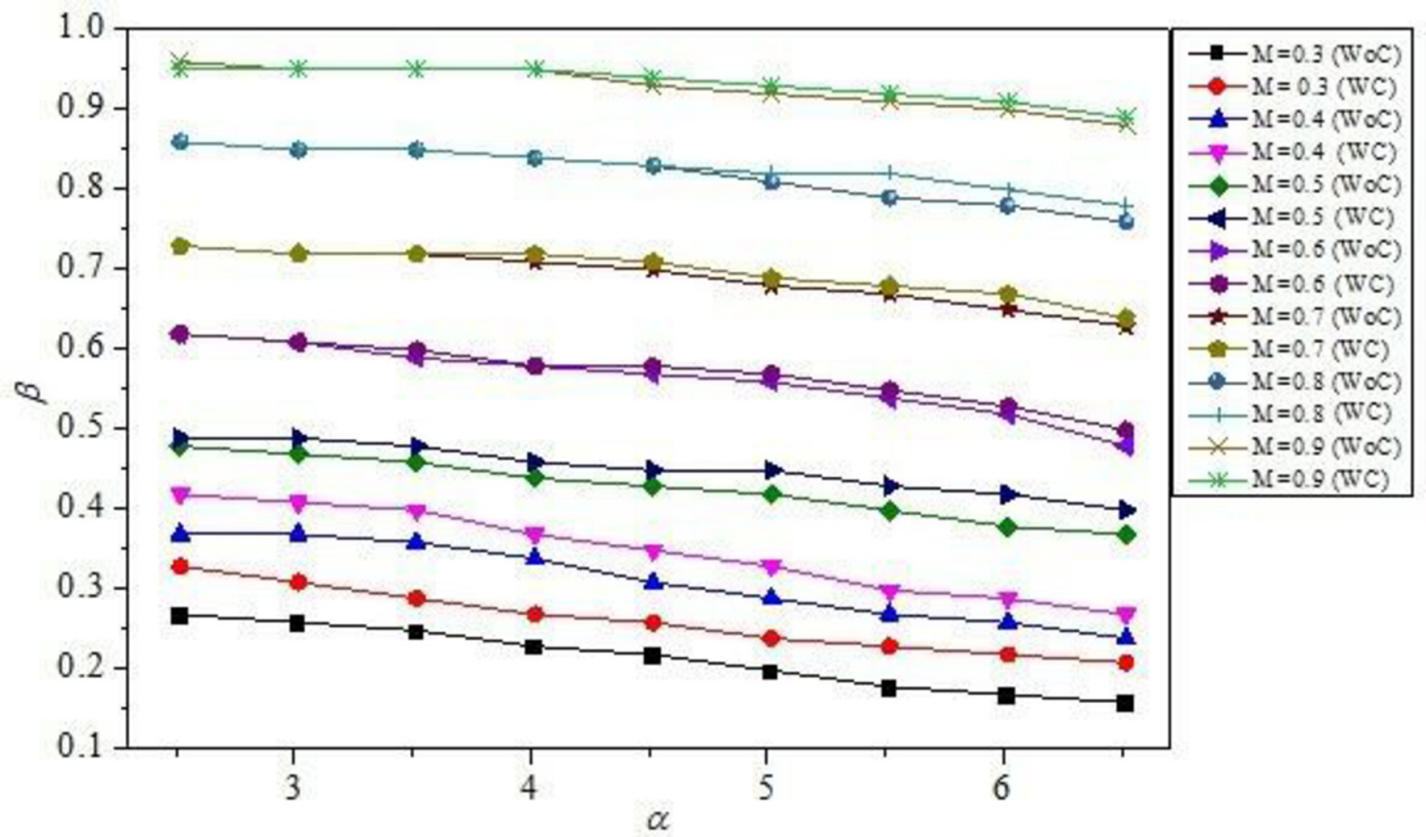
Variation of *β* w.r.t *α* for without control (WC) and with control (WC) for different *M* = 0.3, 0.4, 0.5, 0.6, 0.7, 0.8, and 0.9 for fixed *γ* = 8 and *η* = 1.5.

**Fig 9:**
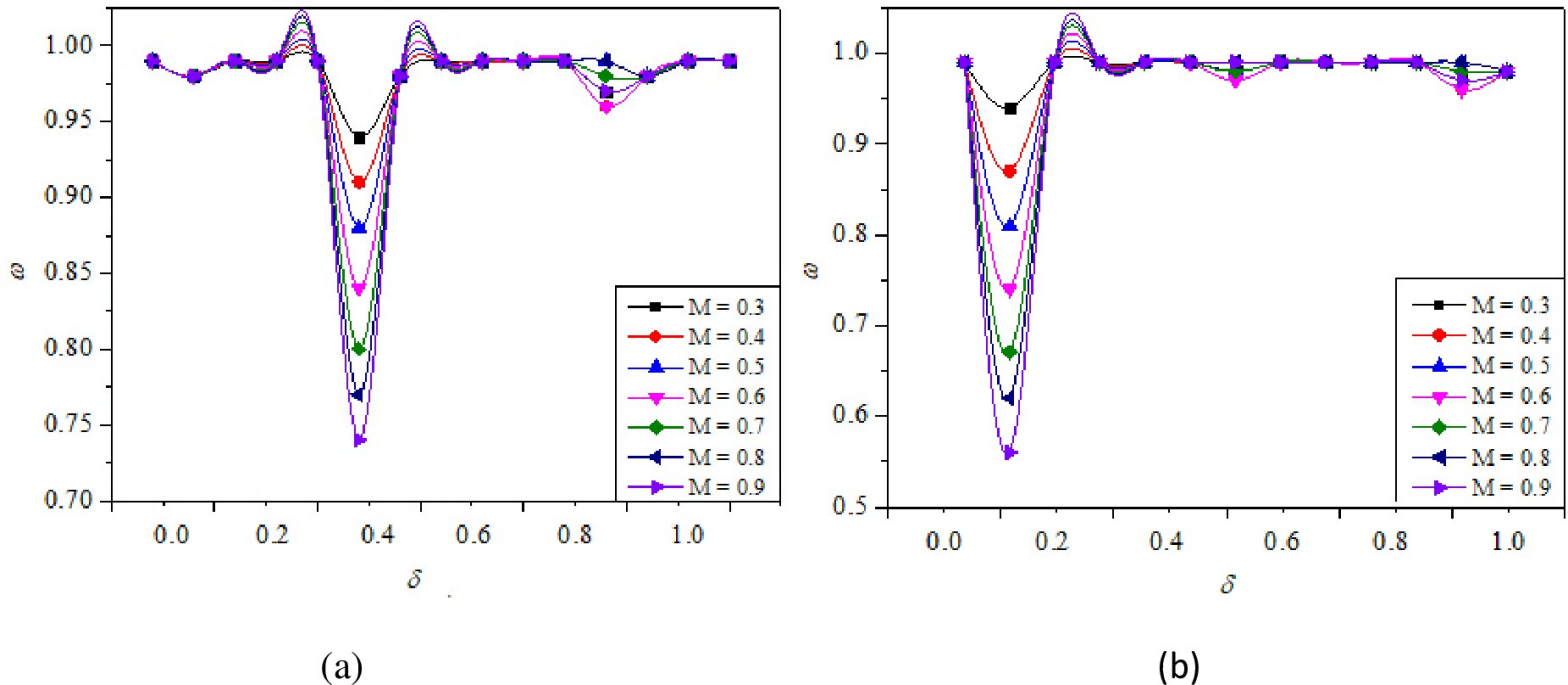
Variation of *ω* w.r.t *δ* for *M* = 0.3, 0.4, 0.5, 0.6, 0.7, 0.8, and 0.9 for constant *η* = 1.5, *α* = 4.75 for (a) *γ* = 7, (b) *γ* = 4.

Fig 8 depicts the variation of *β* for *M* values ranging from 0.3 to 0.9, for *γ* = 2, 6, and 10, for *α* = 4.75 and *η* = 1.5. As can be seen, the behavior of *β* is radically different for smaller values of *γ*, more precisely for *γ* = 2, when compared to higher values. In this case, *β* was seen to decrease as *M* increased. It is critical to note that the shear layers’ reattachment to the nozzle and the subsequent expansion of the boundary layer in the expanded duct are heavily impacted by *γ*. At *γ* = 2, the duct length is inadequate to reattach the flow. Here, the boundary layer near the nozzle exit interacts with the atmospheric pressure, making expansion of the shear layer implausible. As a result, when *γ* is small, *β* is substantially influenced by atmospheric pressure. Fig 9 illustrates the variation of *β* for *α* values ranging from 2.55 to 6.5 and *M* values ranging from 0.3 to 0.9. The values of *α* have a significant effect on the position of flow reattachment points along the expanded duct. A low value of *α* indicates that the flow has very little room to expand, resulting in a low *β* value. As the value of *α* increases, the duct provides enough area for rapid growth, resulting in an increase in *β*. It is critical to notice that as *α* decreases, the boundary layer has very little room to relax, causing the shear layer to expand near the nozzle exit [2, 3]. Increases in *α* have the effect of relaxing the shear layer, causing it to adhere to the larger duct wall. Due to the availability of area for the flow to expand, this shear layer is comparably thicker, and the recirculation flow is projected to be more stable, resulting in higher suction and lower *β*. As a result, *α* is critical in defining the size of the recirculation zone, which in turn affects the suction levels at the base. Additionally, it was determined that the control mechanism is successful for greater values of *α*, i.e., for *α* ≥ 4, and for the whole range of *M* values. Control was ineffective for the combined cases of *M* ≥ 0.5 and *α* ≤ 4. However, as *α* increased above 4, when the control mechanism was deployed, narrow changes in *β* were detected. These variations in *β* were attributed to the relief provided for the flow to expand and the disturbance generated by the control when the vortices in the recirculation zone at the base, shed significantly [1, 12]. Control has been observed to greatly increase *β* for M≤ 0.5, and for the complete range of *α* values. This is because the flow in this range is more irregular and unsteady than in the prior scenario, and is frequently near atmospheric pressure levels [31, 33].

### 4.2. Wall pressure (ω) analysis

**Fig 10:**
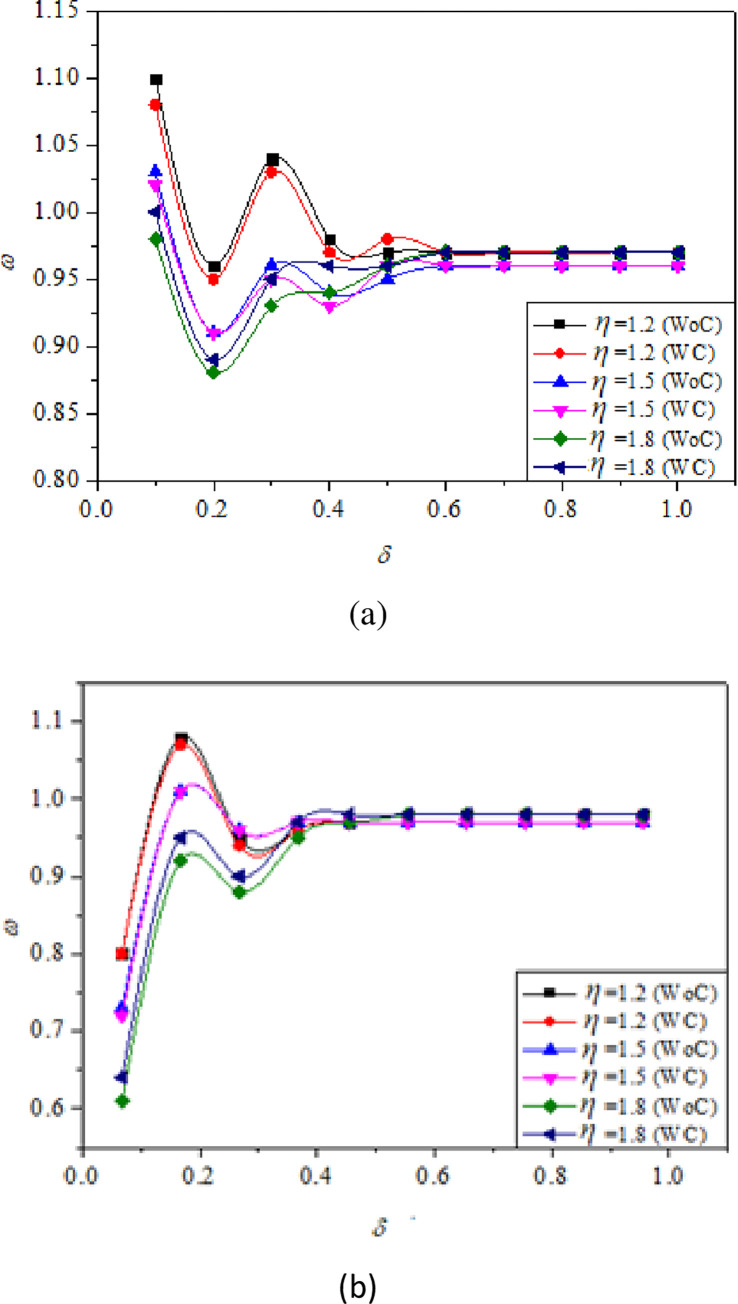
Variation of *ω* with respect to *δ* without control (WC) and with control (WC) for different *η =* 1.2, 1.5, and 1.8 at constant *M* = 0.6, *α* = 4.75 for (a) *γ* = 6, (b) *γ* = 10.

**Fig 11:**
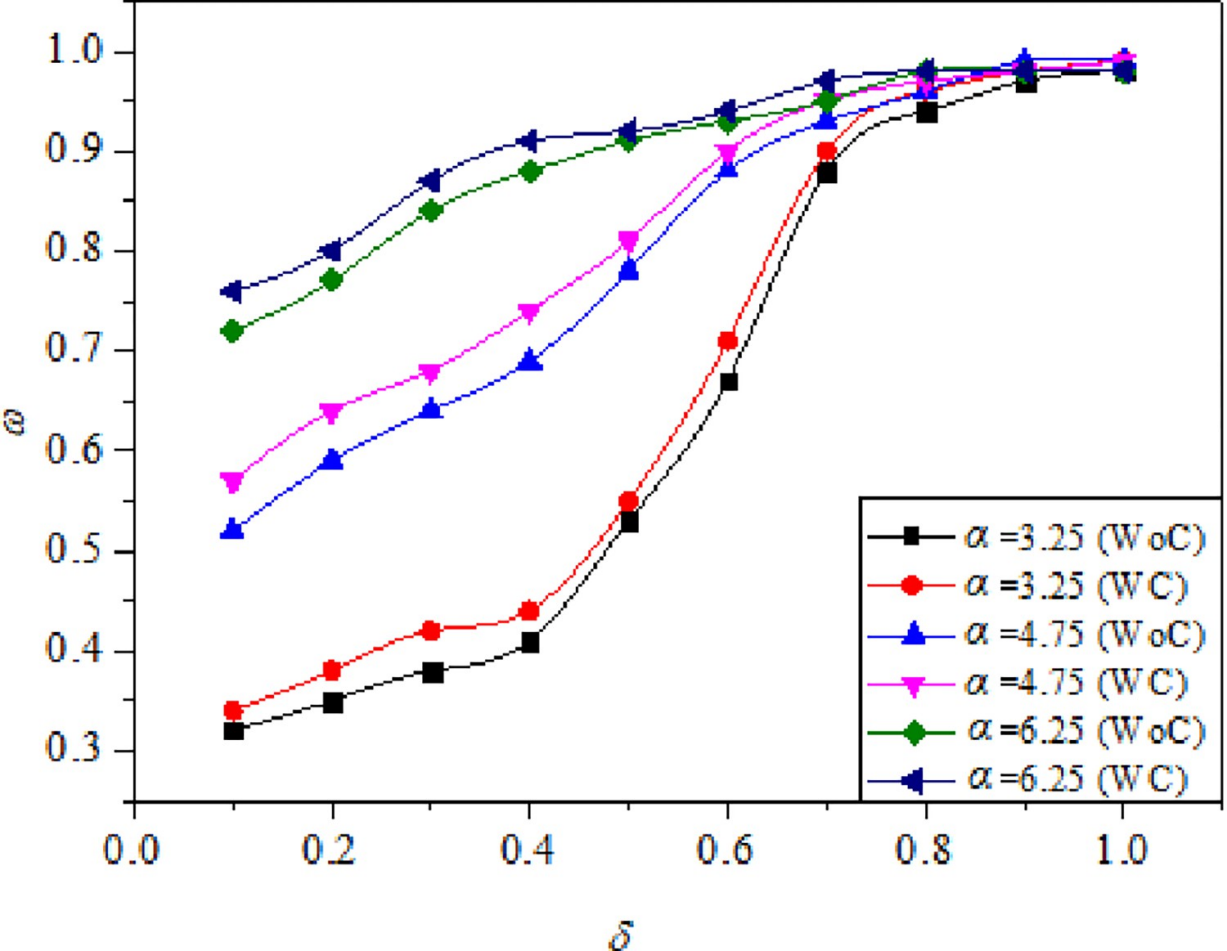
Variation of *ω* with respect to *δ* without control (WC) and with control (WC) for different α = 3.25, 4.75, and 6.25 at constant *M* = 0.6, *η* = 1.5, and *γ* = 8.

At the base region, vortices are generated due to the expansion of the shear layer from the nozzle exit, and are repeatedly discharged into the main flow. Wick [40] termed this action as “Jet Pump-action”. This action causes irregularities and oscillations in the duct flow. These oscillations are evident in changes that take place in the *ω* distribution within the expanded duct. As a result, it becomes vital for a researcher studying abrupt expansion flows to examine *ω* distributions and the evolution of the flow in the expanded duct. When the flow expands from the nozzle into the duct, it expands and intersects with the duct wall and subsequently, compresses in the direction of the flow. Compression and expansion of the flow result in a rise and decrease in pressure along the duct wall, as shown by the *ω* (Figs 9–11).

Fig 9 shows the plots of *ω* w.r.t *δ* for different *M* values for *γ* = 7 and 4. For this particular case, the *α* and *η* were maintained constant at 4.75 and 1.5, respectively, providing adequate space for the flow to expand. The *ω* distribution is straightforward, with a single point of reattachment after which the *ω* equals ambient atmospheric pressure. The reattachment point is very clearly visible at *δ* ≅ 0.4 and *δ* ≅ 0.1, for *γ* = 7, and *γ* = 4, respectively (Fig 9A & 9B). This demonstrates that the distance required for the flow to reattach is significantly smaller for low values of *γ* than for higher values. The large decrease in *ω* represents major reattachment that occurs much earlier in the duct location. Apart from this, there are two or more further small reattachments that are attributable to minor aftereffects of the initial reattachment at *δ* ≅ 0.4 and *δ* ≅ 0.1. The *ω* is shown to decrease dramatically as Mach numbers increase, owing to a stable recirculation zone that generates high suction.

Fig 10 shows the plots of *ω* versus *δ* for *η* = 1.2, 1.5, and 1.8, *γ* = 6, 10. For this particular case, the *α* and *M* were maintained constant at 4.75 and 0.6, respectively. From Fig 10A, the reattachment point is distinctively seen at *δ* ≅ 0.6, for all NPRs and the *ω* is seen to reduce drastically when the *η* was increased from 1.2 to 1.8, as the flow is more stable and the vortex generated is comparatively smaller [12]. The recirculation region is smaller for higher values of *η* as a result of which, higher suction is created at the base and the breakdown of large-scale vortices to small-scale eddies does not completely happen [1]. Similarly, when the *γ* was 10 (Fig 9B), the reattachment point was seen at *δ* ≅ 0.4, which is clearly lesser than the previous case. Afzal et al. [31] reports that for higher values of *γ*, the influence of atmospheric pressure on flow development is minimum resulting in the curbing of flow oscillations at a lower duct location. It can be clearly observed that the flow field is oscillatory for lower values of *γ* as compared to the higher ones. This result is supported by the works of Khan and Rathakrishnan [3, 4]. It is observed that for *ω* along the duct for under-expanded jets, the flow field remains identical for with and without control cases, except, at the duct entrance due to the presence of Mach waves as they generate significant oscillations. As a result, *ω* for both scenarios of under-expanded subsonic flows, the characteristic of the flow remained oscillatory till the flow reattached. This is one of the primary benefits of dynamic controls: they do not exacerbate the flow field in the duct and maintain its uniformity.

Fig 11 illustrates *ω* versus *δ* for α = 3.25, 4.75, and 6.25 at constant *M* = 0.6, *η* = 1.5, and *γ* = 8. It is seen that when we compare the findings for higher α i.e., 4.75 and 6.25 with the lower α value of 3.25 with and without control, there is a slight difference in *ω* distribution along the duct. It can be seen that microjet control has a limited effect for lower values of α. However, as α was raised, it was discovered that control was successful delivering greater values for *ω*. According to Aabid et al. [36], a high α yields the highest significant mass flow rate of air at *M* = 0.6 because of the available space. For α = 3.25 at *η* = 1.5, the flow rate is reduced due to the inability of the area in the duct to provide relief for air expansion. As a result, the net flow rate will fall; hence, for *η* = 1.5, the less relief for the flow is not acceptable. Therefore, when the area is lowered, the control mechanism is unable to influence the flow field due to the decreased net flow rate. At a duct position of *δ =* 0.8, the *ω* was similar to atmospheric pressure.

### 4.3 ANN analysis

**Fig 12:**
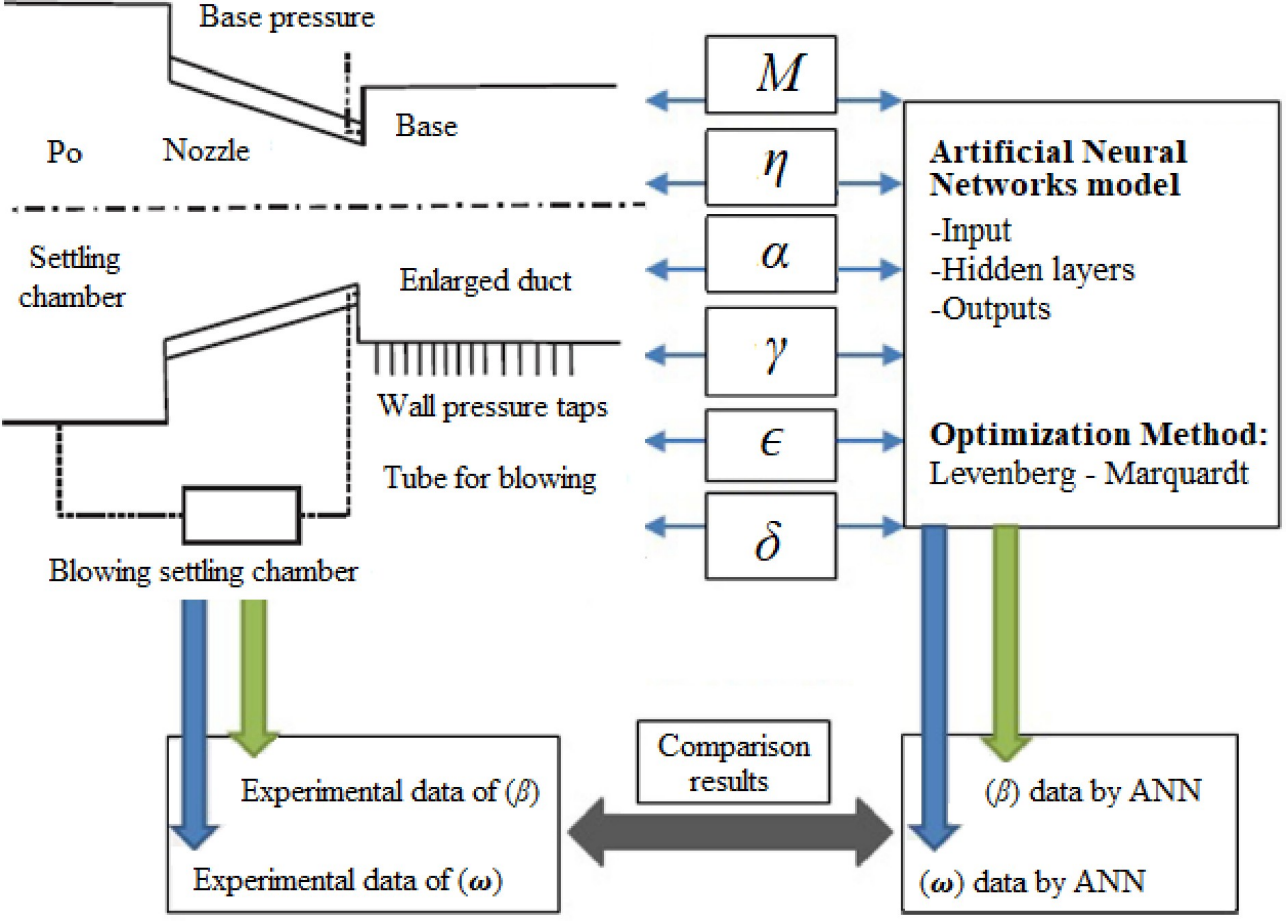
Network of input and outputs used in the study.

To develop a NN model for the expanded flow process, set of experiments were conducted for various input output combinations. A total of 75 combinations retrieved from Figs 6–11 were developed and divided into two sets of which, one set of 60 were used for training, and the second set of 15 were used for testing. The NN model employed a multilayer feed-forward network, tangential sigmoid (TANSIG) function in the hidden layer, back propagation algorithm to search for optimal configuration network using the MATLAB toolbox. The NN model was operated using six inputs and two outputs, as shown in Fig 12. The Levenberg-Marquardt (TRAINLM) algorithm [32] is the quickest training procedure and is employed to modify the weight coefficients and biases for optimization of the network performance function. The various parameters that the ANN model uses during training are mentioned in Table 2. The NN prediction performance was determined using regression analysis through comparison between the predicted parameters and experimental values. *MSE*, *RMSE*, *R*^*2*^, and *MRE* were utilized to evaluate the NN performance. The NN models operated in real time. Intel’s 8^th^ Generation i7 CPU with four cores, eight threads, and a clock speed of 4.2 GHz provided the processing power necessary for real-time functioning. The RAM was 8 GB in capacity and operated at a speed of 2666 MHz. The factors and values used in this investigation are already listed in Table 1.

**Table 2:** ANN model parameters for training.

Parameters	Values
Algorithm for training	TRAINLM
Performance function	MSE
Transfer function	TANSIG
Performance goal	0
Minimum performance gradient	1e-8
Initial value of mu	0.001
Factor for decreasing mu	0.1
Factor for increasing mu	10
Maximum training time (sec)	Inf

#### 4.3.1 Sensitive parameter analysis

To achieve an optimum network, the experimental data obtained is divided into training and testing phases. The first is known as training phase. It is used to calculate the gradient and to update the NN weights and biases in order to reduce the performance function. The process through which the NN learns and identifies the link between its inputs and outputs is called training. This operation is carried out by calculating the difference between the output and the target value. The error achieved is then supplied back into the network, with weights and biases modified to minimize the *MSE*. The second phase is the testing phase, which normally occurs after the training phase. Typically, the testing phase is performed to demonstrate the approval of the NN model. Two configurations were created and utilized to estimate *β* and *ω*. The number of neurons in the hidden layer were determined by multiple experiments to decide the ideal training network.

**Fig 13:**
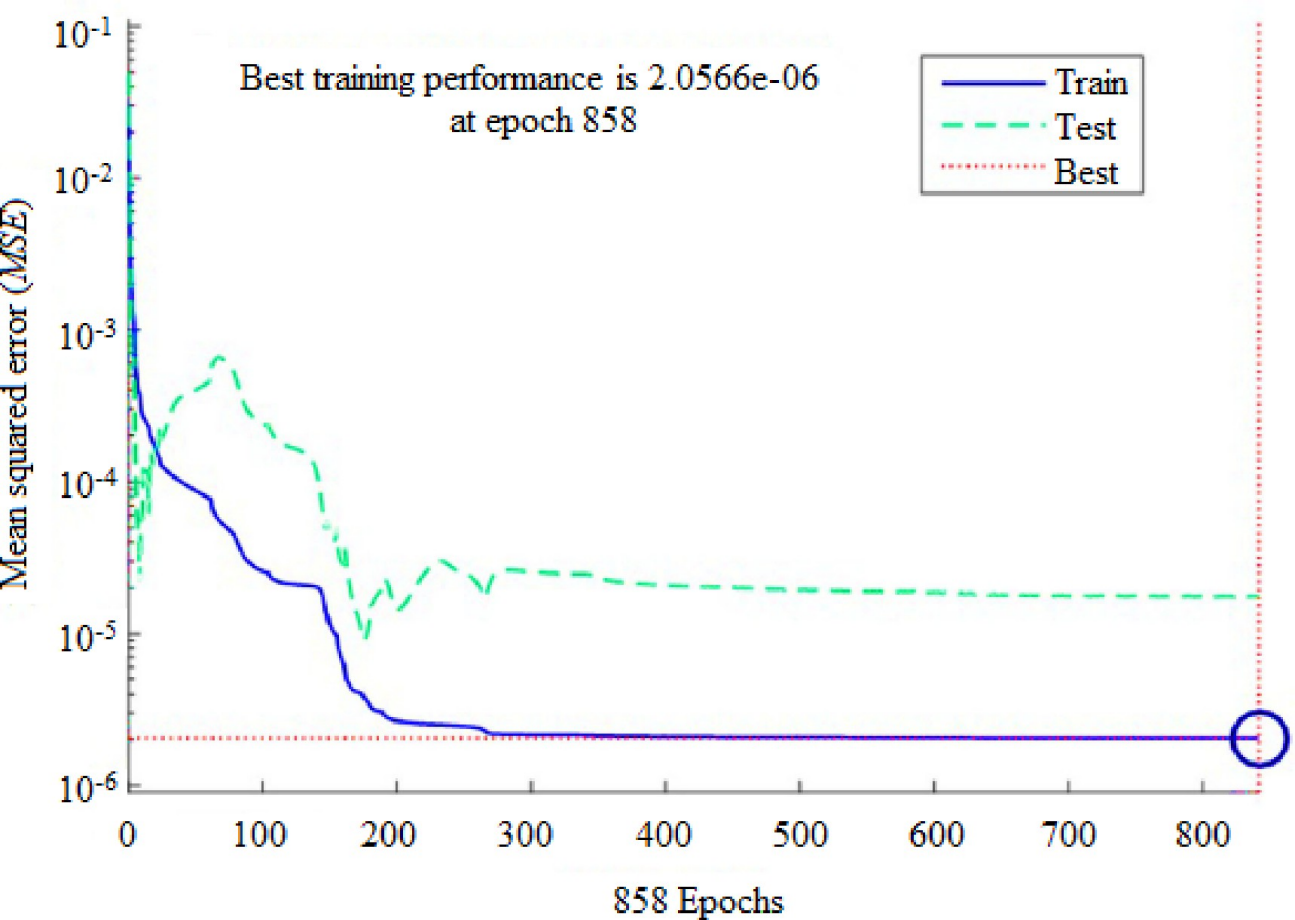
ANN testing and training result for (*β*) based on the 6-5-1 network architecture.

**Fig 14:**
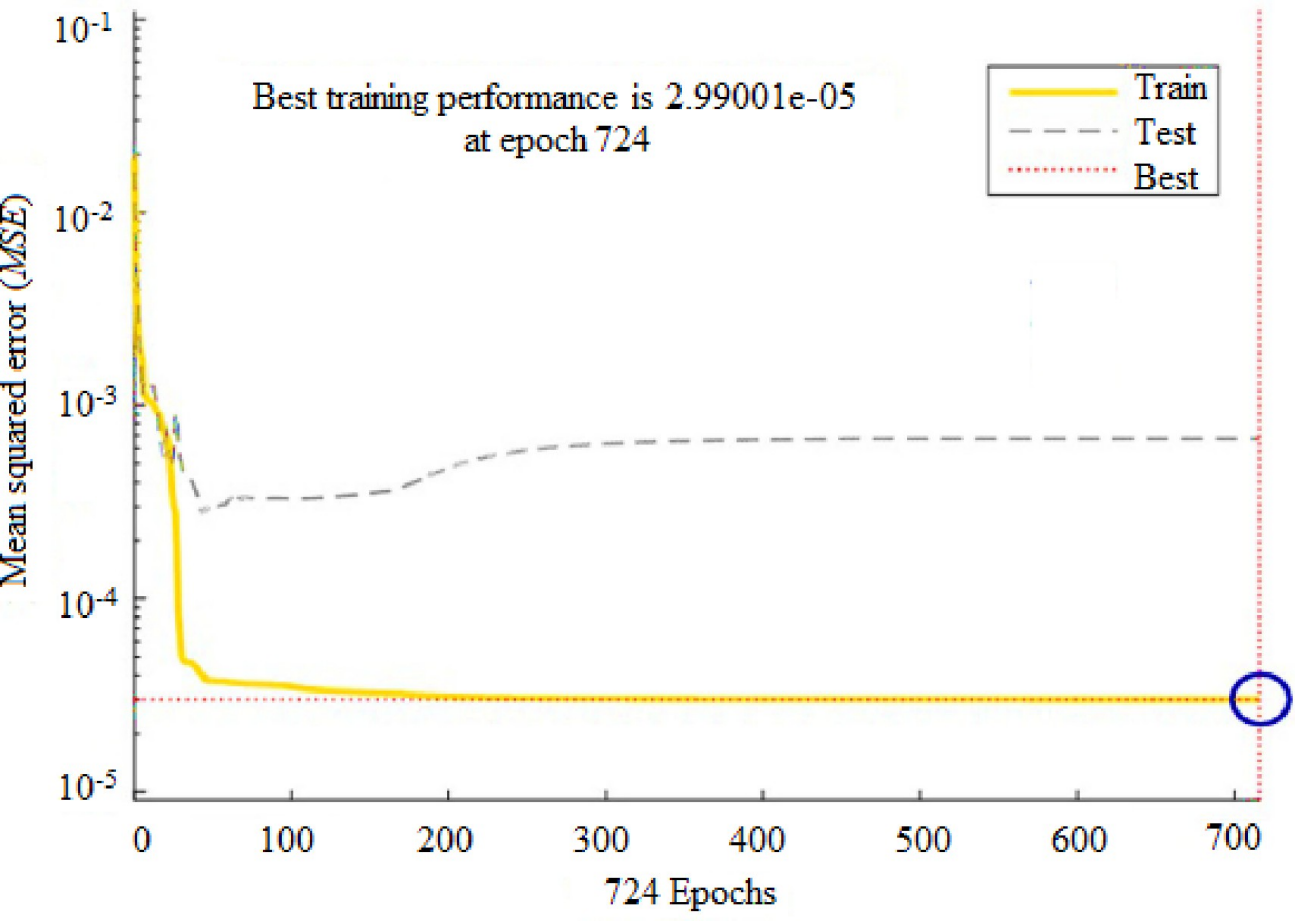
ANN testing and training result for (*ω*) based on the 6-4-1 network architecture.

**Fig 15:**
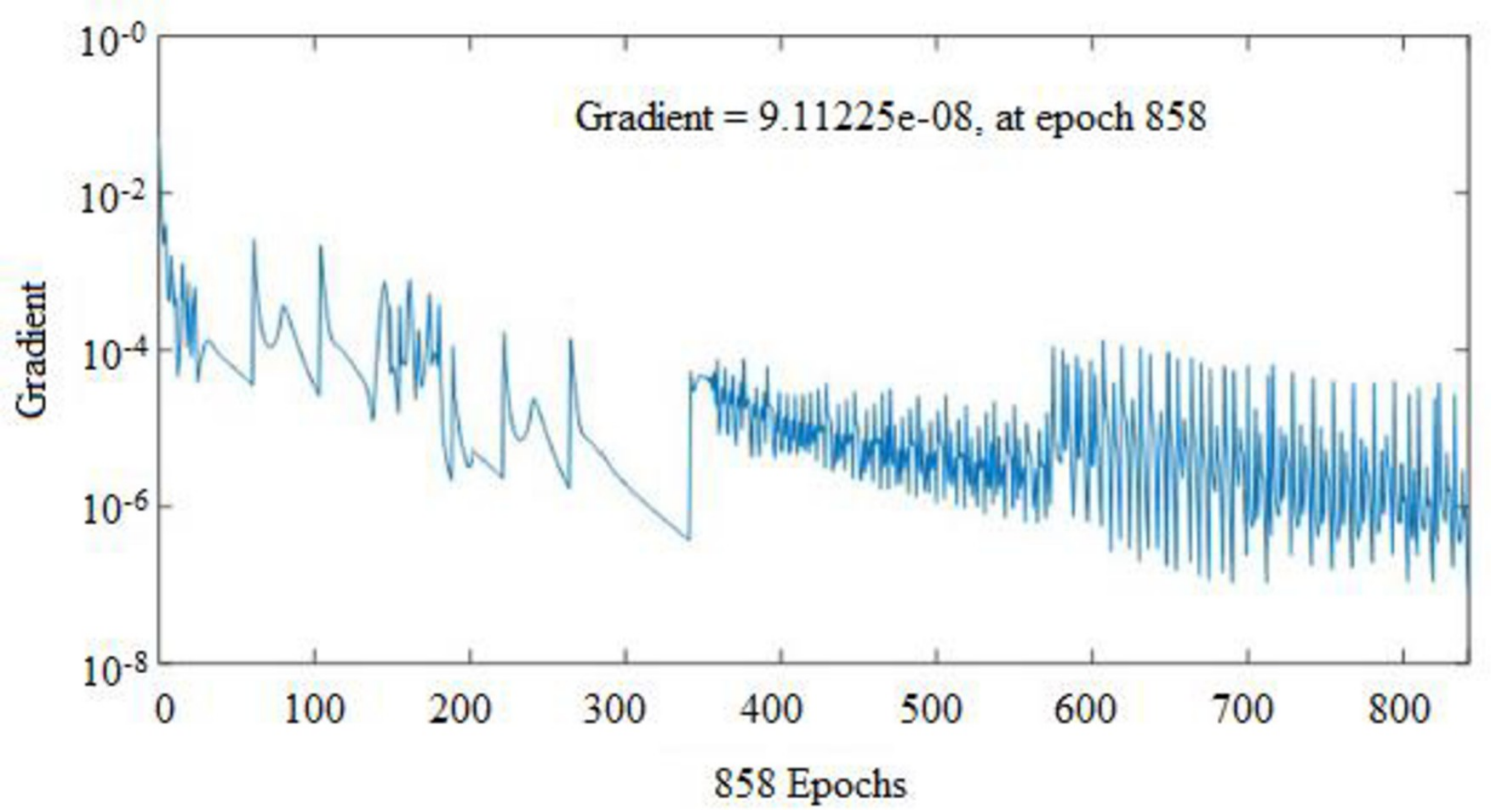
Gradient plot for the (*β*) network output.

**Fig 16:**
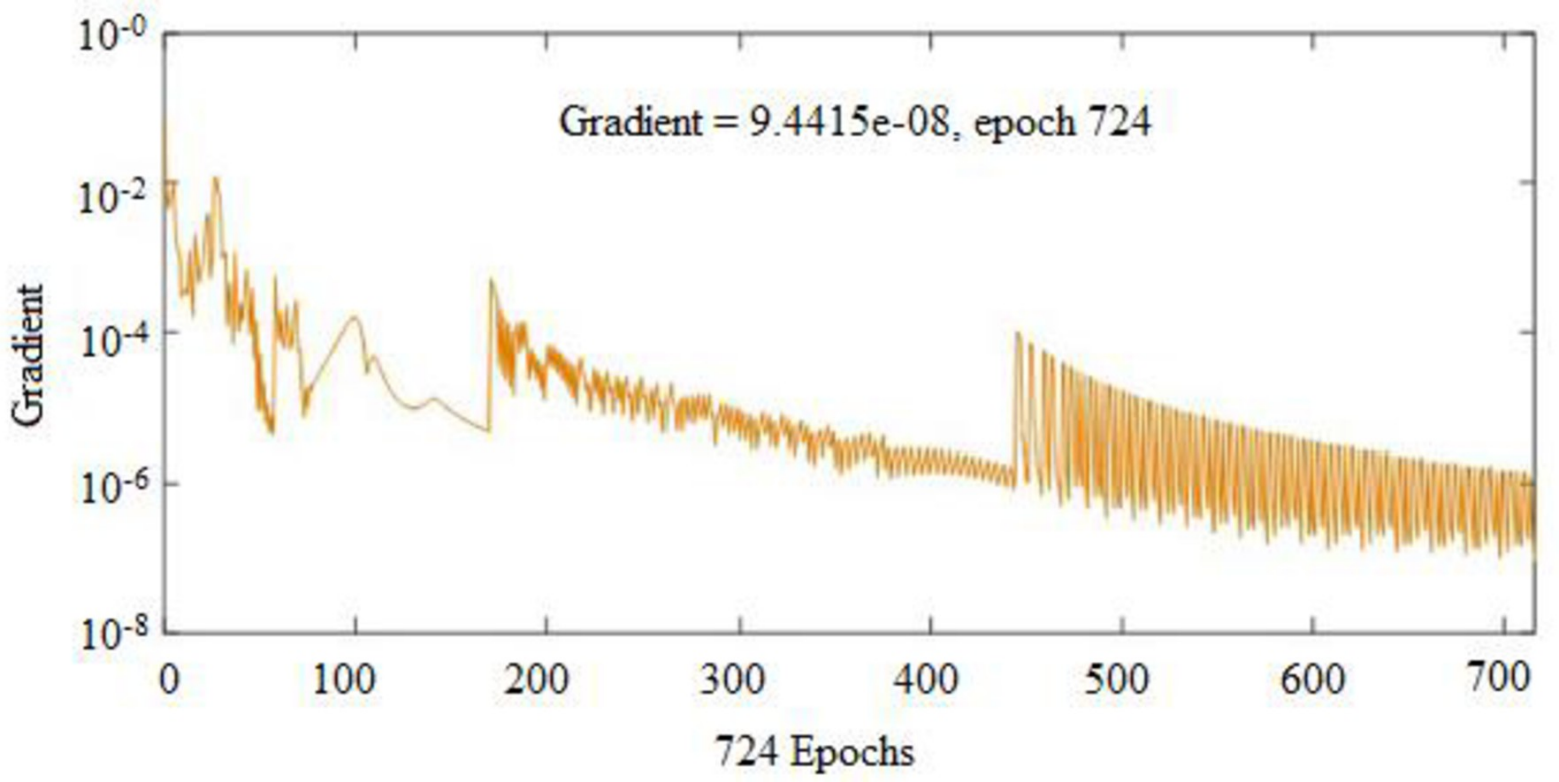
Gradient plot for the (*ω*) network output.

To forecast *β*, the first ANN structure used had six input variables and five neurons in hidden layer (6-5-1). For prediction of *ω*, the second configuration used a network that comprised of six inputs and four neurons in a hidden layer (6-4-1). The ANN prediction and experimental results for the *β* resulted in *R*^*2*^ = 0.99943 and *MRE* = 1.0192% for the training phase, and 0.99676 and 4.7899% for the testing phase, as shown in Table 3. Similarly, the ANN prediction of the *ω* produced *R*^*2*^ = 0.99660 and *MRE* = 0.8190% for the training phase. The Figs 13 and 14 illustrate the emergence of mean squared errors (*MSE*) as a function of the number of iterations (epochs) of the *β* and *ω* for the training and testing phases, respectively. The training was terminated after 858 and 724 iterations for *β* and *ω*, respectively, since the amplitude of the gradient for *β* and *ω* was 9.11225e–08 and 9.4415e–08, respectively (see Figs 15 and 16). Table 3 lists the statistical parameters of the ANN model throughout the training and testing phase for *β* and *ω*.

**Table 3:** ANN performance results for *β* and *ω*.

	Base pressure (*β*)		Wall pressure (*ω*)	
Network structure	6-5-1		6-4-1	
ANN performance	Training	Testing	Training	Testing
*MSE*	2.0566e-06	1.8572e-05	2.99001e-05	5.99003e-05
*RMSE*	0.0021	0.0049	0.0064	0.0305
*MRE (%)*	1.0192	4.7899	0.8190	3.5250
*R* ^*2*^	0.99943	0.99676	0.99660	0.94290
*Training Epoch*	858		724	
*Gradient*	9.11225e-08		9.4415e-08	

**Fig 17:**
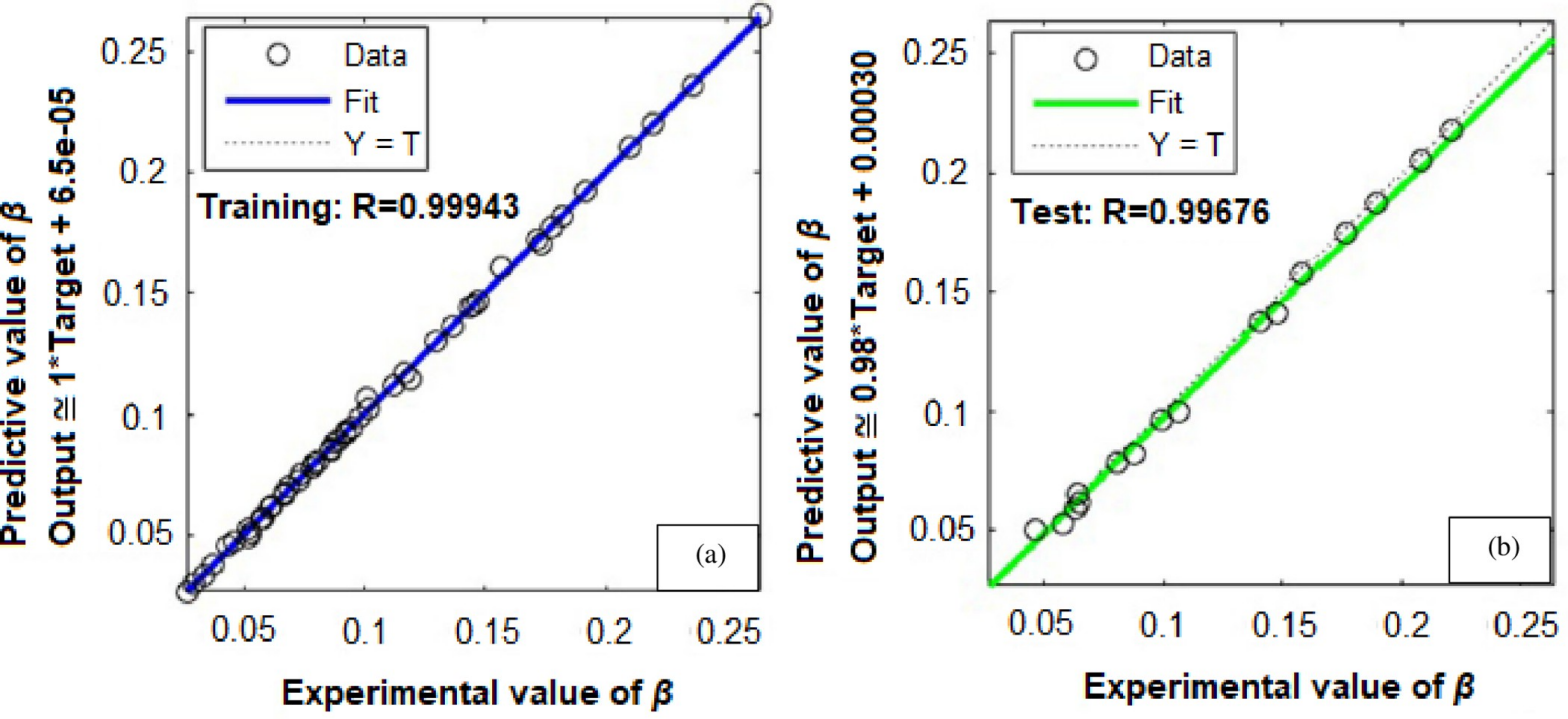
Regression coefficient (*R*^*2*^) for (*β*)—(a) Training data, (b) Testing data.

**Fig 18:**
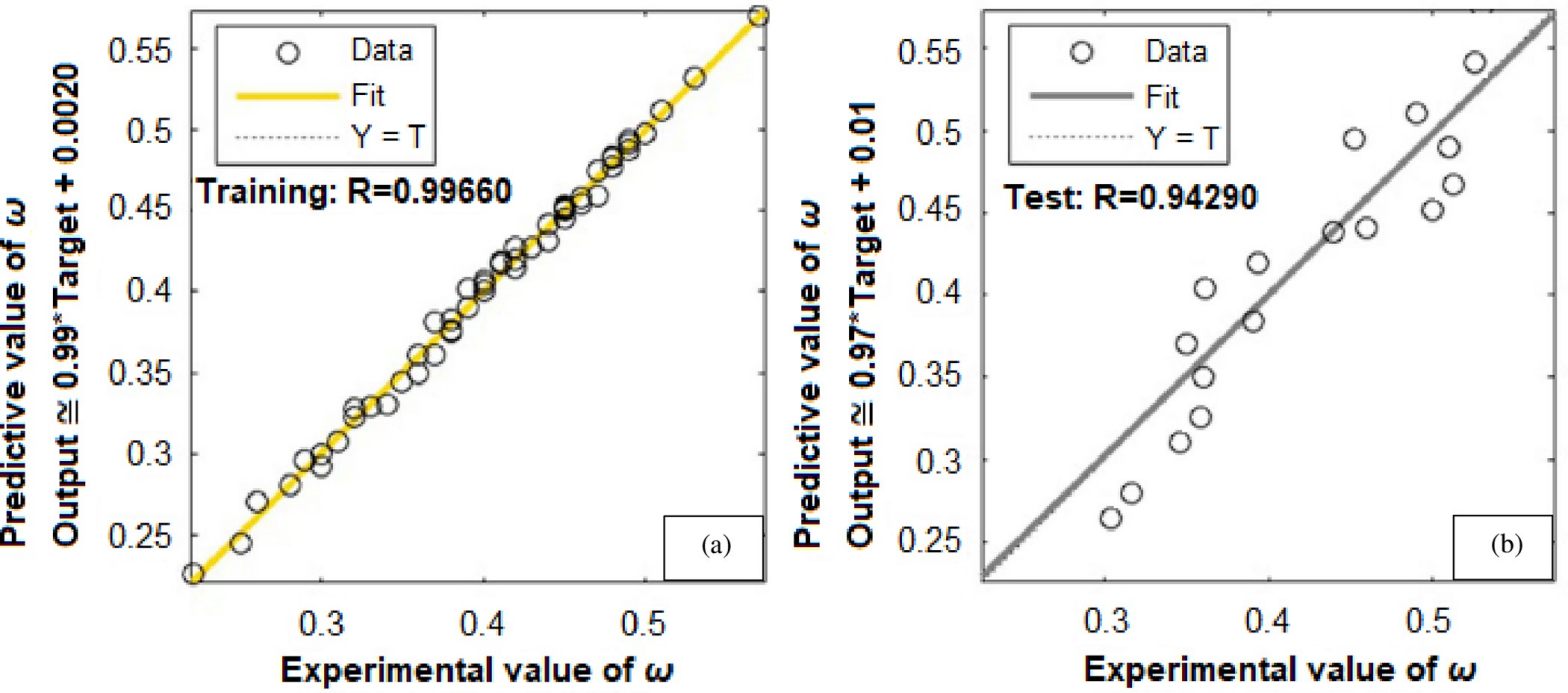
Regression coefficient (*R*^*2*^) for (*ω*)—(a) Training data, (b) Testing data.

A satisfactory decrease in the *MSE* of the statistical parameters was observed to about 2.0566e-06 and 2.99001e-05 for *β* and *ω*, respectively, indicating that the weights are upgraded and the network is formed. Figs 17 and 18 shows the plots for (*R*^*2*^) wherein, the data from ANN simulations are compared with experimental data. The *R*^*2*^ values achieved for *β* and *ω* during the training and testing phases of the ANN configurations (6-5-1) and (6-4-1) were entirely within the acceptable range, equating to a lower *MSE* and higher (*R*^*2*^) values. The predicted *β* values demonstrated a very high correlation (*R*^*2*^) of 0.99943 for training, and 0.99676 for testing. Similarly, for *ω* the (*R*^*2*^) was determined to be 0.99660 for training and 0.94290 for testing. These findings suggest that the ANN model may be effectively utilized to forecast the *β* and *ω* in a suddenly expanded subsonic flow process.

### 4.3.2. Comparison of experimental and ANN results

**Fig 19:**
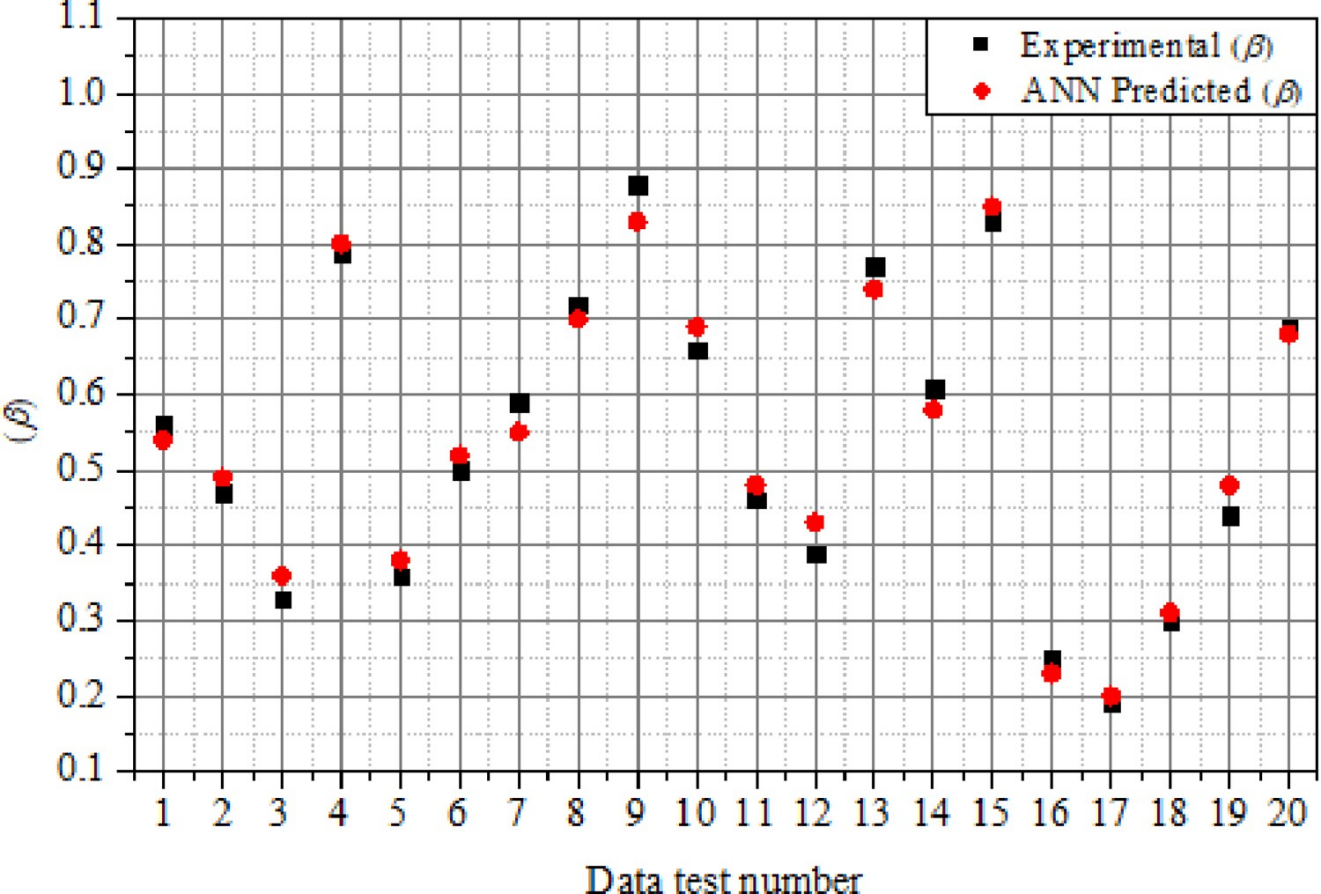
Comparison of experimental and ANN predicted results for (*β*).

**Fig 20:**
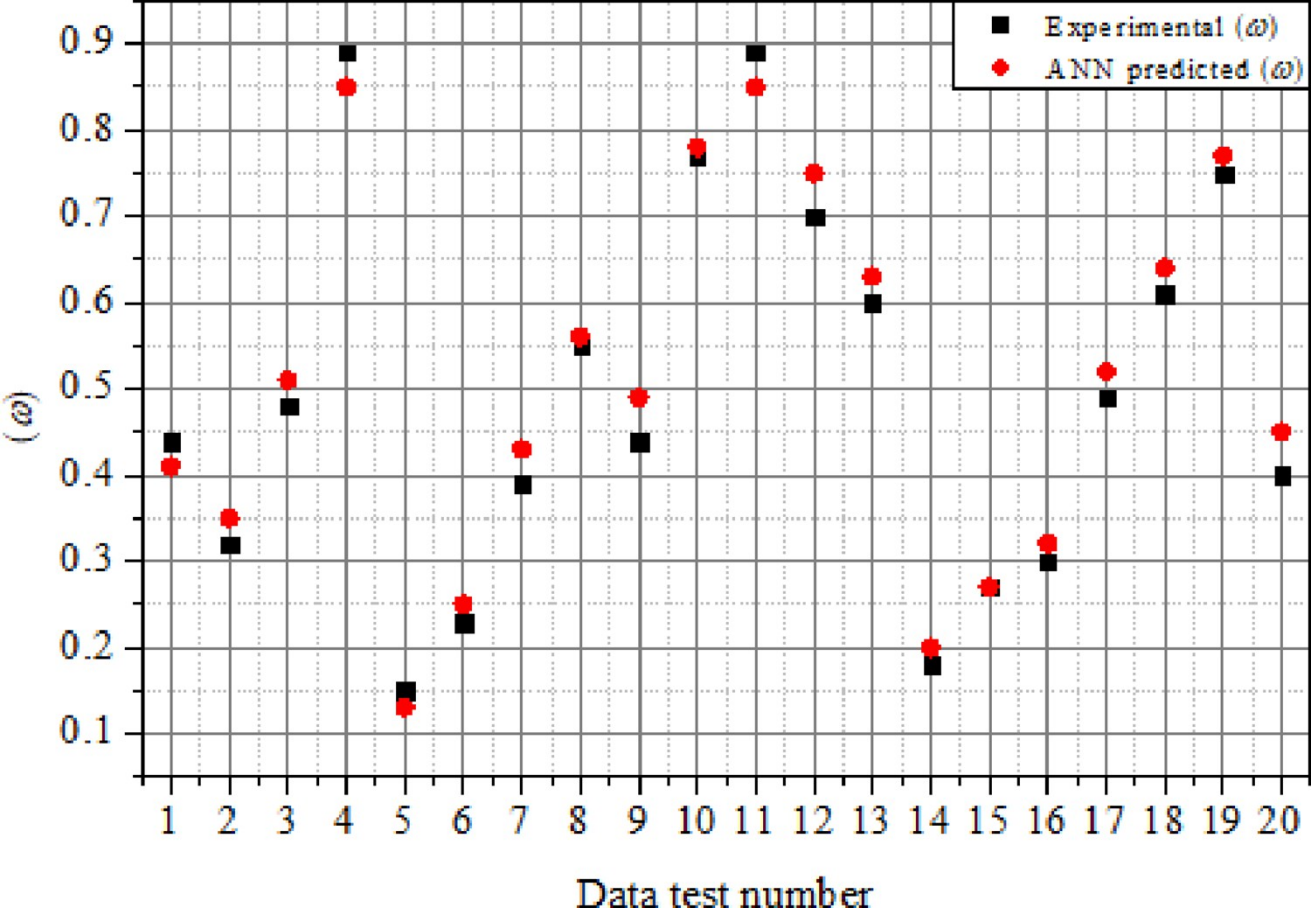
Comparison of experimental and ANN predicted results for (*ω*).

The simulated *β* results were matched with the experimental data in order to determine the reliability and accuracy of the ANN model. Figs 19 and 20 illustrate the outcomes of the comparison between the prediction values generated using the ANN model and experimental data of *β* and *ω*. The values predicted by the ANN model for *β* were quite close to those observed in the experiment, as seen in Fig 19. The range of relative error (*ER*_*r*_%) computed using Eq 12 was in the range of 1.266% to 10.25%, indicating a strong agreement between the experimental and ANN predicted values. Likewise, as seen in Fig 20, the values forecasted by the ANN model for *ω* were in good agreement with the experiment results. The range of relative error (*ER*_*r*_%) obtained was between 1.299% to 13.33%, indicating good agreement between the ANN predictions and experimental findings. Table 4 compares the simulation results of the ANN model to the experimental data of the suddenly expanded subsonic process as defined by the *β* and *ω*. As can be observed, the ANN-based percentage error is far lower, and the ANN model findings demonstrated good accuracy when compared to experimental data.

$${ER}_{r}(\%)=\frac{\left|Q_{exp}-Q_{ANN}\right|}{Q_{exp}}\times 100$$ (12)

*Q*_*ANN*_ represents the ANN predicted value, and *Q*_*exp*_ represents the experimental value.

**Table 4:** Experimental and ANN predicted result comparison for *β* and *ω*.

Base pressure (*β*)			Wall pressure (*ω*)		
Experimental value	ANN predicted value	Difference *ER*_*r*_ *(%)*	Experimental value	ANN predicted value	Difference*ER*_*r*_ *(*%*)*
0.56	0.54	3.571	0.44	0.41	6.819
0.47	0.49	4.256	0.32	0.35	9.375
0.33	0.36	9.090	0.48	0.51	6.250
0.79	0.80	1.266	0.89	0.85	4.494
0.36	0.38	5.556	0.15	0.13	13.33
0.50	0.52	4.000	0.23	0.25	8.696
0.59	0.55	6.780	0.39	0.43	10.25
0.72	0.70	2.778	0.55	0.56	1.819
0.88	0.83	5.681	0.44	0.49	11.36
0.66	0.69	4.546	0.77	0.78	1.299
0.46	0.48	4.348	0.89	0.85	4.494
0.39	0.43	10.25	0.70	0.75	7.143
0.77	0.74	3.897	0.60	0.63	5.000
0.61	0.58	4.920	0.18	0.20	11.11
0.83	0.85	2.410	0.27	0.26	3.703
0.25	0.23	8.000	0.30	0.32	6.667
0.19	0.20	5.263	0.49	0.52	6.122
0.30	0.31	3.333	0.61	0.64	4.919
0.44	0.48	9.090	0.75	0.77	2.667
0.69	0.68	1.449	0.40	0.45	12.50

Additionally, Willmott [41] presented the statistical parameter Index of Agreement (*IA*) as a measure for ANN model effectiveness. If (*IA* = 1), there is no difference between the experimental and the predicted values. Eq 13 gives the IA (0 ≤ *IA* ≤ 1) [41]:

$$IA=\frac{\sum_{i=1}^{n}{\left(Q_{i,ANN}-Q_{i,exp}\right)}^{2}}{\sum_{i=1}^{n}{\left(\left|Q_{i,ANN}-{\bar{Q}}_{exp}|+|Q_{i,exp}-{\bar{Q}}_{exp}\right|\right)}^{2}}$$ (13)

where, *Q*_*i*,*ANN*_ represents the ANN predicted value, *Q*_*i*,*exp*_ is the experimental value, and ${\bar{Q}}_{exp}$ is the mean experimental values.

The results indicate that the *IA* values for *β* and *ω* output networks were 0.996 and 0.942, respectively. As a result, it is revealed that the ANN-predicted results are in good conformity with the experimental data. Additionally, Table 5 compares the outcomes of this study to the earlier research on ANNs that were performed for flows of supersonic Mach range. *MSE* and *R*^*2*^ values are great numerical indicators of prediction accuracy of the NN model. A well-trained ANN model generally has a low MSE and a high *R*^*2*^ value. In comparison to the other studies mentioned in Table 5, the *β* derived from the NN with a 6-5-1 structure gave an acceptable degree of accuracy with *MSE* = 1.8572e-05 and *R*^*2*^ = 0.99676. Similarly, the *ω* produced from the NN with a 6-4-1 structure was also accurate (*MSE* = 5.99003e-05 and *R*^*2*^ = 0.94290), demonstrating the validity of the ANN technique for simulating a suddenly expanded subsonic flow process. As a result, the input variables employed in the current NN model have a significant influence on the output prediction and should be investigated in order to optimize the subsonic flow process.

**Table 5:** Comparison the literature with the present study.

Literature	Mach number range	Network structure	Output neurons	*MSE* for best performance	*R*^*2*^ (Testing)
Afzal et al. [31]	Supersonic	4-4-1	Base pressure (*β*)	0.0235	-
		4-4-4-1	Wall pressure (*ω*)		
		4-5-1			
		4-5-5-1			
		4-6-1			
		4-6-6-1			
Quadros et al. [32]	Supersonic	3-6-1	Base pressure (*β*)	0.032	<0.99
Quadros et al. [42]	Supersonic	4-9-9-1	Base pressure (*β*)	0.01872	0.9295
				0.000453	0.8931
		4-9-9-1			
Present study	Subsonic	6-5-1, 6-4-1	Base pressure (*β*)	1.8572e-05	0.99676
			Wall pressure (*ω*)	5.99003e-05	0.94290

#### 4.3.3 Sensitivity of input parameters

To determine the sensitivities of the input factors on the suddenly expanded flow process, an expression provided by Garson [43] was employed, as mentioned by Hernandez *et al*. [44], Hamzaoui *et al*. [45], and Reyes-Téllez *et al*. [46]. This method was later improved by Goh [47]. The proposed equation is based on weighted connection between the input layer and hidden layer, as well as between the hidden layer and the output layer. Thus, Eq 14 was stated as follows for the relative importance (*I*_*j*_ %) [47]:

$$I_{j}(\%)=\frac{\sum_{m=1}^{Nh}\left(\left(\frac{\left|W_{jm}^{i,h}\right|}{\sum_{n=1}^{Ni}\left|W_{nm}^{i,h}\right|})\times |W_{ml}^{h,o}\right|\right)}{\sum_{n=1}^{Ni}\left[\sum_{m=1}^{Nh}(\frac{\left|W_{nm}^{i,h}\right|}{\sum_{n=1}^{Ni}\left|W_{nm}^{i,h}\right|})\times |W_{ml}^{h,o}|\right]}$$ (14)

where, *I*_*j*_ is the relative importance of the *j*^*th*^ input parameter on the output, *N*_*i*_ and *N*_*h*_ represent the input and hidden neurons, respectively, and *W* is the weight connection. The subscripts *i*, *h*, and *o*, represent input, hidden, and output; *n*, *m*, and *l* represent the neuron numbers in the input, hidden, and output layers, respectively.

**Fig 21:**
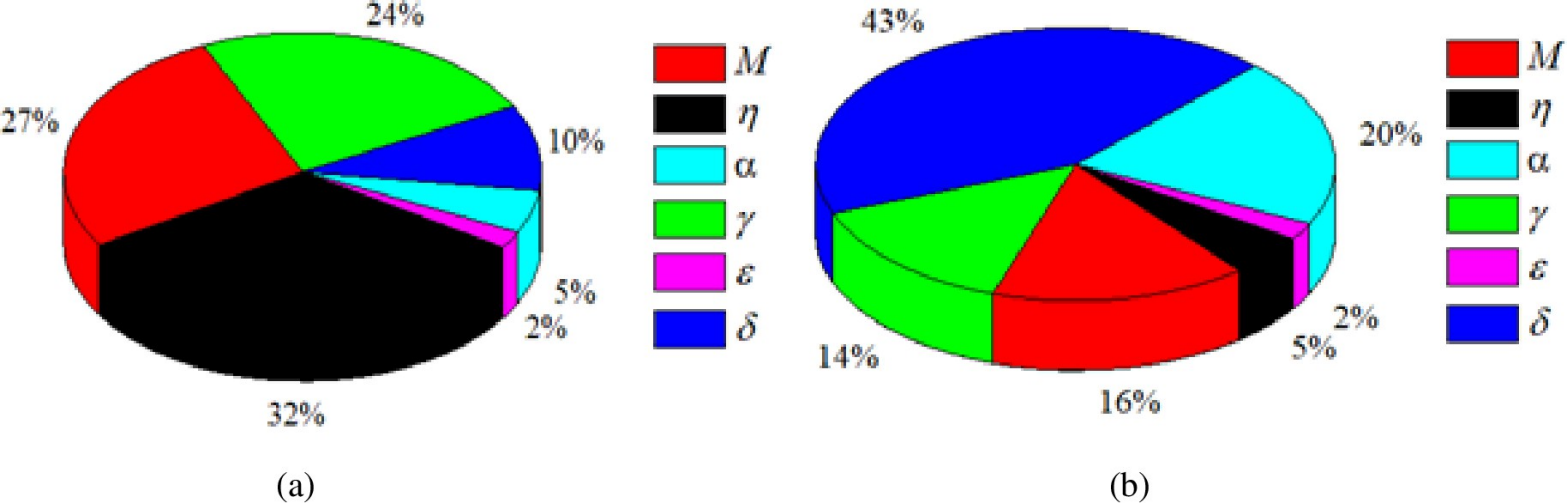
Sensitivity of input variable for (a) *β* and (b) *ω*.

The weight and bias values of the current ANN analysis for base pressure and wall pressure have been provided in Tables 6 and 7, respectively. Also, when Equation 18 is used, the operating parameters have a significant influence on the flow process, as seen in Fig 21. Fig 21A & 21B show the relative importance of all six input variables such as, *M*, *η*, *α*, *γ*, ϵ, and *δ* on the output *β* and *ω*. It can be observed that all variables had some effect on *β*. First, the input parameter *η* was determined to have the highest influence of 32% on *β*. Second, M and *γ* showed significant influence of 27% and 24%, respectively. Furthermore, the effect of *δ* was notable to a certain extent with 10%, and *α* and ϵ had the least influence on *β* with 5% and 2%, respectively. The reason behind the high influence of *η* was a result of formation of small recirculation zones for varying values of *η*. This eventually altered the suction strength at the base, thereby breaking the large-scale vortices into small-scale eddies. Likewise, Fig 21(B) demonstrates the relative importance of the input variables on *ω*. It was observed that *δ* had the highest influence on *ω* with 43%, followed by *α* with 20%, M with 16%, *γ* with 14%, *η* and *ϵ* with 5% and 2%, respectively. The high influence of *δ* was due to the influence of atmospheric pressure on flow development resulting in the of flow oscillations for varying duct locations.

**Table 6:** Weights and biases for the ANN model (*β*).

*W*_*ij*_	Hidden neurons: *j* (1,. . . 5)				
Input *i* (1, … 6)	3.3884	0.0341	-2.2971	-0.0079	-0.0288
	1.9568	0.6641	-1.1180	0.3478	-2.7840
	-9.2245	1.8801	8.4400	1.0567	4.8671
	-0.0256	-0.2045	0.1890	-0.1081	0.7187
	7.7912	1.8844	-0.9242	1.6590	-1.9231
	7.2350	4.4540	0.3421	3.4340	-0.1330
*W*_*1j*_ (k = 1)	1.5670	22.679	1.4480	-70.128	0.4056
b1_(*k*)_	-7.0046	-8.2055	-5.6545	-6.7901	2.0563
b2_(s)_	-39.445				

**Table 7:** Weights and biases for the ANN model (*ω*).

*W*_*ij*_	Hidden neurons: *j* (1,. . . 4)			
Input *i* (1, … 6)	-0.8077	0.9041	-0.1201	-1.9450
	30.883	-18.776	-0.8830	3.2545
	-13.867	18.325	0.6074	1.9245
	0.7340	-3.0191	0.0183	-3.0521
	-1.1320	20.469	-3.1023	0.7514
	22.661	-10.454	1.6750	0.6626
*W*_*1j*_ (k = 1)	-0.5035	1.2841	32.705	-0.7512
b1_(*k*)_	-32.225	-10.681	2.9715	0.6501
b2_(s)_	-27.545			

**Fig 22:**
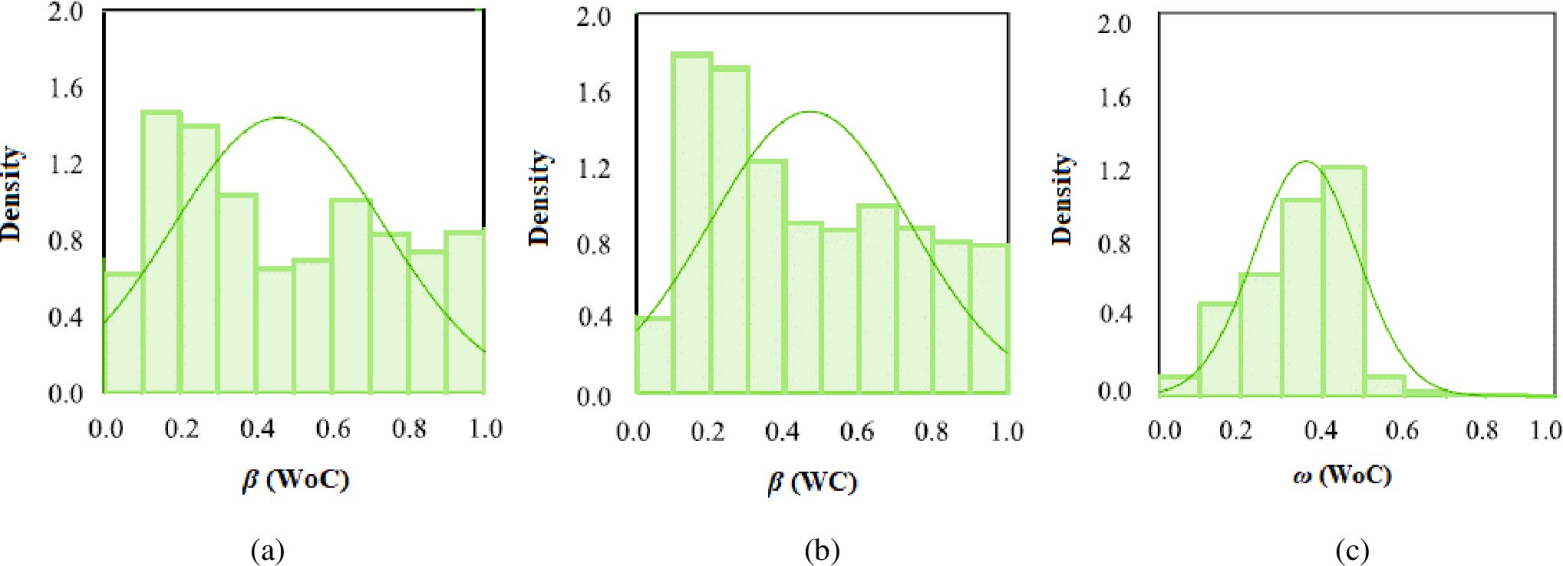
Histogram plot of the *β* for the case of (a)WoC, (b) WC, and (c) *ω* (WoC).

The histogram is a tool for visualizing the dispersion of data that is large in number. Due to the considerable size of our experimental data, we chose to include a histogram to determine how active control impacted the *β* and *ω*. The control values were chosen for *ω*, since without control readings were likewise same and had very less effect. As seen in Fig 22, the pressure is spread evenly across 10 classes ranging from 0 to 1. The density of a class reflects its density. Although *β* histograms appear identical, a closer examination reveals that the density is greater for *β* (WC) than (WoC) in the 0.2–0.3 and 0.3–0.4 ranges (Fig 22A & 22B). This implies that for certain pressure classes, the intensity of flow WoC is less, demonstrating that active control through micro-jets gives a surge to increase the *β*. As seen in Fig 22(A) and 22(B), the density is greater for WC than for WoC. This implies that the *β* is greater when the flow is in the subsonic regime, due active control. Fig 22(C) illustrates the *ω* density. The pressure class with a value between 0.3 and 0.5 has the maximum density and is about twice as dense as the other classes. As a result, the wall pressure is huge for just this class, and the density is poor for pressures more than 0.6.

## 5. Conclusions

Experiments have been conducted to develop an ANN-based model, to forecast the suddenly expanded subsonic flow process in terms of *β* and *ω*. Also, the Garson approach was utilized to determine the critical operating parameters affecting the flow process. The following are the major conclusions that can be made from the findings of the ANN model:

- Increased values of *M* formed a stable recirculation zone, resulting in a low *β*. When the value of *η* was increased, a strong suction at the base was created and the breakdown of large-scale vortices into small-scale eddies did not occur completely, resulting in low *β* value.
- For a low value of *α*, the boundary layer had very little room to relax, resulting in an expansion of the shear layer near the nozzle exit. Increase in *α* caused the shear layer to relax, allowing it to attach with the larger duct wall. Due to the available space for the flow to expand, this shear layer became significantly thicker, and the recirculation flow was predicted to be more stable, resulting in a low *β*.
- A low *γ* indicated that the boundary layer near the nozzle exit is greatly influenced by ambient pressure, restricting the growth of the shear layer, leading to a significant decrease in the *β* value.
- The variation of *ω* demonstrates that the distance required for the flow to reattach is significantly smaller for low values of *γ* as compared to the higher ones. Moreover, multiple minor reattachments were observed after the major attachment that occur at the end of the duct location. It is also found that for under-expanded jets, the flow field is always same for with and without microjet control, except near the duct entrance, where Mach waves are present and considerable oscillations occur.
- When compared to experimental data, the predicted *β* and *ω* values using the ANN model demonstrated a high degree of accuracy. Correlation coefficients of 0.9996 and 0.9976 were achieved for training and testing, respectively. The same was found for *ω*, which was 0.9947 and 0.9319 for training and testing, respectively.
- The *η* and *δ* were the critical parameters that were most influential *β* and *ω*, with a relative importance of 32% and 43%, respectively.
- The results demonstrate that the ANN model is capable of accurately predicting the suddenly expanded subsonic flow process under current flow conditions, thereby establishing a theoretical foundation for research into pressure distribution in aerodynamic systems, which is critical for developing new design concepts.

## Abbreviations

ANN: Artificial Neural Network
BBD: Box-Behnken design
C-D: convergent divergent
CCD: central composite design
CFD: computational fluid dynamics
D: diameter of the expanded duct, mm
DoE: design of experiments
*ER_r_*: Relative error
*IA*: Index of Agreement
L: length of the expanded duct, mm
L/D: length to diameter ratio
*M*: Mach number
*MRE*: mean relative error
*MSE*: Mean square error
PID: Proportional Integral Differential
PURELIN: linear transfer function
*R^2^*: regression coefficient
RANS: Reynolds Averaged Navier Stokes
*RMSE*: root mean square error
RSM: response surface methodology
TANSIG: tangent sigmoid transfer function
WC: with microjet control / with control
WoC: without microjet control / without control
*Greek symbols α*: area ratio
*β*: non-dimensional base pressure / base pressure
*γ*: length to diameter ratio
*δ*: duct location to length ratio
*ϵ*: microjet control
*η*: nozzle pressure ratio / level of expansion
*ω*: non-dimension wall pressure / wall pressure
*ER_r_*: Relative error
*IA*: Index of Agreement
*Subscripts ANN*: artificial neural networks
*B*: bias values / threshold values
*Exp*: Experimental
*i*, *h*, and *o*: input, hidden, and output, respectively
*j*, *k*, and *s*: Input, hidden, and output layers, respectively
*Max*: Maximum
*Min*: Minimum
*n*, *m*, and *l*: Number of neurons in the input, hidden, and output layers, respectively
*Nor*: Normalized
*Net*: Network
